# Assessing the impact of temporal changes in transmission on *Plasmodium falciparum* strains in Asembo, western Kenya (1996–2017) using within-host metrics via 24-SNP barcodes

**DOI:** 10.1186/s12936-025-05700-3

**Published:** 2025-12-17

**Authors:** Gary Vestal, Zhiyong Zhou, Sheila Sergent, Mili Sheth, Justin Lee, Kephas Otieno, Simon Kariuki, Andrew Hill, Feiko O. ter Kuile, Kim A. Lindblade, Laurence Slutsker, Mary J. Hamel, Meghna Desai, John E. Gimnig, Aaron M. Samuels, Ymir Vigfusson, Ya Ping Shi

**Affiliations:** 1 Department of Computer Science, Emory University, Atlanta, USA; 2 Division of Parasitic Diseases and Malaria, National Center for Emerging and Zoonotic Infectious Diseases, Centers for Disease Control and Prevention, Atlanta, USA; 3 Biotechnology Core Facility Branch, Division of Core Laboratory Services and Response, Office of Laboratory Systems and Response, CDC, Atlanta, USA; 4 Kenya Medical Research Institute, Centre for Global Health Research, Kisumu, Kenya; 5 Department of Clinical Sciences, Liverpool School of Tropical Medicine, Liverpool, UK

## Abstract

**Background:**

Genomic surveillance of malaria parasites offers important insights into the impact of interventions on transmission reduction and changes in pathogen populations over time, especially in low-transmission areas. However, such surveillance faces challenges in high-transmission regions. Detecting temporal changes in transmission in high-transmission settings requires analytical methods tailored to high-diversity parasite populations that can differentiate between superinfection (infection through multiple mosquito bites, each bearing an unrelated strain) and co-transmission (infection through a single mosquito bite bearing more than one strain).

**Methods:**

This study applied a previously developed novel Next Generation Sequencing (NGS) 24-SNP barcode assay for genotyping smear-positive samples obtained from a 2017 cross-sectional survey in the Asembo area, western Kenya, building on previous work on samples collected in the surveys conducted in 1996, 2001, 2007, and 2012. Algorithms StrainRecon and STIM were used to identify parasite strains within a sample and measure multiplicity of infection (MOI). Population genetic metrics of *F*_*ST*_ (Fixation Index), strain-relatedness by IBD (Identity by Descent), *H*_*s*_ (Modified Heterozygosity) and *N*_*e*_ (Effective Population Size) were evaluated using the same 24-SNP data. This study further explored a novel slope metric of the relationship between within-host strain relatedness and MOI to infer superinfection and co-transmission. Temporal changes in the above metrics were assessed.

**Results:**

There was no significant differentiation in* F*_*ST*_, *H*_*s*_*, N*_*e*_*,* and strain-relatedness at the population level over time. In contrast, the average MOI significantly decreased from 4.32 in 1996 to 3.34 in 2012, although it increased to 3.49 in 2017. Insecticide-treated bednet distribution campaigns from 1997 to 2017 did track these temporal changes in MOI. Additionally, the value of strain relatedness within-host (IBD) was inversely correlated with MOI (number of strains), and the change in the inverse relationship (within-host slopes) over time was verified by two different correlation analysis and modelling. The temporal trends in this within-host slope metric suggested that transmission dynamics shifted towards co-transmission from 2001 to 2012, and then returned to similar levels of superinfection in 2017 as in 1996.

**Conclusion:**

The within-host MOI, IBD-based strain-relatedness, and their mathematical relationship (slope) provide useful metrics for understanding the transmission dynamics in our study. Notably, this study presents the first simple slope-based method using 24-SNP barcodes to distinguish superinfection from co-transmission in a high transmission area, warranting further evaluation of the novel tool in other high transmission settings.

**Supplementary Information:**

The online version contains supplementary material available at 10.1186/s12936-025-05700-3.

## Background

Over the last two decades, malaria control interventions in the Asembo area of western Kenya, a high transmission area, have significantly reduced malaria transmission [1]. This progress has been achieved largely by scaling up vector control interventions to reduce transmission and increasing access to effective antimalarial treatment for case management [2]. The impact of these control efforts has been mainly assessed using traditional methods, such as monitoring infection prevalence and entomological inoculation rate (EIR) [3, 4]. However, parasite population genomic surveillance has increasingly been used to estimate transmission intensity, particularly in areas with low transmission levels [5].

Recent studies have shown the operational utility of single nucleotide polymorphism (SNP)-based genomic surveillance in diverse African settings. Schaffner et al. [5] successfully implemented national-scale surveillance in Senegal using 24-SNP barcodes, analyzing over 1,000 samples from clinical cases from 23 health facilities and demonstrating that polygenomic infection proportions serve as reliable proxies for transmission intensity [5]. Watson et al*.* [6] developed predictive transmission models including using 24-SNP barcodes and showing that molecular surveillance could be a novel surveillance tool for estimating the prevalence of malaria in areas in which prevalence surveys are not feasible. Their models revealed that genetic metrics can accurately predict transmission intensity across different epidemiological settings. Together, these studies emphasize the practical implementation of 24-SNP barcode surveillance at scale and suggest its potential utility as a complementary approach to traditional epidemiological monitoring.

One particularly useful genetic metric is multiplicity of infection (MOI), which is defined as the number of concurrent distinct genotypes/strains of parasites within a host. MOI is positively associated with transmission intensity [7–9], making it an opportune metric for analysing changes in transmission across different time periods and locations. Such analyses have been performed on genome-wide SNP and 24 SNP barcode data [10–13], including in the Asembo area in western Kenya, using multi-locus neutral microsatellite markers (MS) [14, 15] and, most recently, strain barcodes of 24 genome-wide SNPs [13].

MOI is increased by two different transmission processes. In superinfection, the patient is infected through multiple mosquito bites each bearing a unique and unrelated parasite genotype/strain. In co-transmission, the patient is infected through a single mosquito bite bearing more than one parasite genotype/strain [16, 17]. Strains in superinfections tend to be less related than those in co-transmission within the host [16, 17]. Understanding whether complex infections arise from superinfection or co-transmission or both is essential for accurately interpreting transmission dynamics [11, 16, 17], as superinfection directly reflects the frequency of infectious mosquito bites [18], whereas co-transmission can occur even with declining mosquito-to-human transmission, making traditional MOI measurement potentially misleading as sole genetic indicator of transmission intensity [19]. This distinction has become particularly important following a demonstration by Nkhoma et al. [16] that co-transmission dominates even in high-transmission settings in Malawi, challenging previous assumptions regarding complex infections.

Furthermore, MOI is subject to various confounding factors. The resolution, sensitivity, and accuracy of MOI measurements are influenced by the study methods used, including the choice of genetic/genomic markers as well as methods for genotyping, data processing, and analysis. MOI measurements can further be confounded by factors within each host, such as host immunity (age) and parasite density, and presence or lack of symptoms, particularly when antigen gene markers are used [20–22]. These variables may limit the MOI as a standalone metric for surveillance.

Another core notion in malaria parasite population genetics is strain-relatedness, both between different populations and within individual hosts. The classical fixation index (*F*_*ST*_), which measures population differentiation due to genetic structure [23], has been applied at temporal and large geographical scales using both MS and SNP data. However, *F*_*ST*_ is uninformative at smaller spatial scales or at the within-host level [24]. Recent analyses have instead applied the concept of identity by descent (IBD) [25]. IBD measures the variability in strains that are broken down by recombination, thus relating this variability to shared ancestry. Thus, IBD can measure parasite strain-relatedness both between human populations and at the within-host level. IBD has been used in conjunction with 24 SNP barcodes to monitor transmission intensity at temporal and fine spatial scales in low transmission areas of Senegal [19]. More importantly, an IBD analysis of within-host *Plasmodium falciparum* strain-relatedness using whole-genome sequencing (WGS) data and 24 SNP data in the low-transmission area of Senegal revealed that co-transmission is a major contributor to polyclonal infections [19]. In addition, analysis of WGS data from single cloned parasites from the high malaria transmission area of Malawi showed that co-transmission predominantly shapes within-host parasite diversity, contradicting the conventional assumption that superinfection would be predominant in high transmission areas [16]. Overall, IBD-based relatedness analysis provides a powerful and high-resolution tool for studying the genetic relatedness of malaria parasites at the within-host level although systematic underestimation has been observed at population level [26].

Transmission intensity can also be estimated by other population genetics metrics, including expected heterozygosity (*H*_*e*_), which assesses genetic diversity within parasite populations, and effective population size (*N*_*e*_), which estimates the uneven reproductive success of the parasite population’s individual lineages [27, 28]. *H*_*e*_ has been positively associated with transmission intensity on a large geographic scale [10]. A reduction in the* N*_*e*_ is expected if transmission is reduced [29], and it has been applied in the analysis of *P. falciparum* MS and SNP data, mainly in relatively low transmission areas [11, 30]. However, these metrics have limitations. In studies in high-transmission areas based on MS and genome-wide SNP signature data, *H*_*e*_ has not provided sufficient resolution for inferring temporal changes in transmission intensity [10, 14, 15]. Meanwhile,* N*_*e*_ is subject to several confounding factors, such as migration, sample size, and calculation method [31].

Despite these advances in low transmission settings, genomic surveillance of the temporospatial dynamics of malaria parasites at the population and micro levels remains particularly challenging in medium- and high-transmission areas. In these areas, simultaneous infection with multiple malaria parasite strains within-host is common [7–9]; however, no method exists for accurately sorting the alleles of these complex infections into their respective strains (haplotypes). Therefore, genomic surveillance-based estimates of transmission intensity in these areas are still in the preliminary stage.

A recent study conducted by this research group addressed these dilemmas using a novel set of high-resolution tools, including advanced next-generation sequencing (NGS)-based *P. falciparum* 24 SNP barcode assay, a unique bioinformatics pipeline for data processing, an algorithm for reconstruction of parasite strains (StrainRecon), and a novel MOI estimator (STIM) [13, 32]. Importantly, the StrainRecon algorithm is designed to reconstruct every strain of sufficient proportion present in a sample, including previously unseen strains [32]. Together, these tools were used to analyse a set of high-MOI field parasites samples collected in 1996, 2001, 2007, and 2012 during cross-sectional population-based surveys in Asembo, western Kenya. The analysis was able to (i) reliably reconstruct the SNP barcodes of strains in individual samples and (ii) detect a decrease in the MOI over time [13].

This study presents the temporal genomic surveillance of *P. falciparum* populations at the same site in western Kenya using NGS-based 24-SNP barcode data. Building on the previous work, this study makes four key contributions: (1) expanding the temporal analysis from 1996 to 2012 [13] to include 2017, capturing potential resurgence; (2) evaluating whether traditional population genetics metrics designed for low transmission area (predominantly clonal infection) remain informative in high-transmission settings; (3) analysing the relationship between MOI and parasite density as well as between MOI and age over time to further validate our molecular tool and analytical pipeline; and (4) introducing a novel within-host metric based on the slope of the IBD-MOI relationship to distinguish trends in superinfection versus co-transmission, addressing a critical gap identified by recent studies. These findings enhance understanding of malaria transmission dynamics in the current study conducted in a high transmission area of western Kenya and provide valuable insights for future malaria genomic surveillance of transmission changes in other medium- and high-transmission areas.

## Methods

### Ethics statement

This temporal study was approved by the Scientific and Ethics Review Unit (SERU) of KEMRI, Nairobi, Kenya and the Institutional Review Board (IRB) of the Centers for Disease Control and Prevention (CDC), Atlanta, USA under different protocol numbers over time. All these study protocols included the consents for blood sample collection and the use of samples for parasite genotyping and analysis. Permission for shipping specimen from Kenya to Atlanta CDC malaria laboratory was granted by the SERU of KEMRI, Nairobi, Kenya.

### Study area and sample collection

This study used the blood samples collected in community-based surveys conducted in Asembo, Siaya County, western Kenya, which is a high-transmission area. These surveys were conducted to evaluate the impact of malaria control interventions on transmission reduction within a Health and Demographic Surveillance System (HDSS) [33]. Malaria vector control began in 1997 with a large-scale trial of insecticide-treated nets (ITNs) conducted within the HDSS area [34, 35]. ITNs were provided to half of the villages in Asembo in 1997, while the remaining villages received nets in 1999. Net re-treatment services every 9–12 months were provided until 2006, after which long-lasting insecticidal nets (LLINs) were used. Nationally, ITNs were initially distributed to children and pregnant women through social marketing in the lake and coastal endemic areas and epidemic prone areas. In 2006, the first mass distribution campaign of LLINs was implemented targeting children under 5 years of age and pregnant women. Subsequent mass campaigns in 2011, 2014, and 2017 targeted all age groups to achieve universal coverage [36, 37]. Bednet usage data in Asembo area were also retrieved from different time points of surveys, 1996 and 2001 from cross-sectional surveys of the ITN trial, 2007 and 2012 from cross sectional surveys of Evaluation of the Introduction of Malaria Control Interventions on Malaria Parasitemia and Anemia (P&A Survey) and 2017 from Malaria Indicator Household Surveys to Evaluate the Impact of Malaria Transmission Reduction Activities: a continuous rotating panel survey (cMIS). Bednet usage is defined as the proportion of people that reported sleeping under a net the previous night.

During the population-based surveys, whole blood samples in 1996, 2001, 2007 and dry blood spot (DBS) samples in 2012 and 2017 were collected. Malaria thick smears were collected from consenting participants in the surveys for quantifying parasites density by microscope. The blood and DBS samples were stored in Kenya in −80 ºC freezers until they were shipped to Atlanta. A total of 372 smear-positive samples were used for 24 SNP testing. Among these, 65 samples from 1996 and 72 samples from 2001 were randomly selected among children aged between 0.4 and 6 years and 53 samples from individuals aged 1–14 years in 2007, 83 samples from individuals aged 0.2–37 years in 2012 and 99 samples from individuals aged between 1-91 years in 2017 were used for this study based on sample availability. Parasite density of study subjects in these five sampling time points from 1996 to 2017 were available and linked to their samples.

### Laboratory testing

Parasite genomic DNA was extracted from the DBS samples using the QIAamp^®^ DNA Mini Kit (QIAGEN, Valencia, CA). Three multiplex PCRs were used to amplify DNA, as previously described [13]. Briefly, multiplex combinations were 7-way (SNPs: 2, 3, 6, 8, 10, 15, 23); 8-way (SNPs: 4, 5, 7, 11, 12, 14, 16, 21) and 9-way (SNPs: 1, 9, 13, 17, 18, 19, 20, 22, 24). Multiplex PCR reactions consisted of 12.5 μl 2 × Platinum Multiplex PCR Super mix (Thermo Fisher Scientific, Waltham, MA, USA), 100 nM each primer, 2 μl DNA template, and PCR water in a 25-μl reaction. The thermal cycling conditions were as follows: 95 °C for 2 min and 35 cycles of 95 °C for 30 s, 60 °C for 1.5 min, and 72 °C for 30 s, with a final extension at 72 °C for 10 min. Laboratory strains, both single strains and mixtures of strains, were employed as positive controls, and normal blood as a negative control was used for each test plate. The PCR products of the three multiplex PCRs were pooled together for each sample and purified on QIA quick PCR purification columns (QIAGEN).

MiSeq Libraries were prepared using a standard 16S Metagenomics sequencing protocol (Illumina, San Diego, CA, USA), as described in a previous iteration of this study [13]. Briefly, PCRs for attaching dual indexing barcodes and adapters were performed with Nextera XT Index Primers. The final libraries were analysed using a Fragment Analyser (Agilent, Santa Clara, CA, USA) for size and quantifying concentration. The libraries for all samples were normalized to 10 nM and pooled at equal volume. The final pool was diluted to 2 nM to prepare for the loading. Up to 54 barcoded samples were pooled in one plate run using the Illumina 2 × 250 bp kit. The sequence reads were filtered for read quality, base called and demultiplexed using bcl2fastq (v2.19). The sequencing results were saved for bioinformatics pipeline data processing purposes.

### Definitions

Definitions of genetic metrics and concepts used in this study are listed in Table 1.

**Table 1:** Definitions of genetic metrics and concepts used in this study

MOI	***Multiplicity of Infection*** (a.k.a. “Complexity of Infection”). The number of concurrent distinct strains of same species of pathogen within a host [50]
StrainRecon	An algorithm for constructing the strains in a sample for a given number of strains *k* [32]
STIM	***StrainRecon Thresholding for Infection Multiplicity.*** An algorithm that uses StrainRecon to estimate the true number of strains *k* in a sample using a predetermined goodness-of-fit threshold [13]
*H*_*e*_	***Expected Heterozygosity.*** A common measure of genetic diversity within populations [27]
*H*_*s*_	A modification of *H*_*e*_ which accounts for sampling imbalances [38]
*F*_*ST*_	***Fixation Index.*** A measure of genetic differentiation between two or more populations due to genetic structure. It ranges from 0 (i.e. no genetic differentiation) to 1 (completely genetic differentiation) [23]
IBD	***Identity by Descent.*** A measure of genetic relatedness between two strains based on estimations of which DNA segments were inherited from a recent common ancestor. It ranges from 0 between two unrelated strains to 1 between identical strains. IBD-above 0.5 generally indicates significant relatedness. The value decreases by recombination [25]
*N*_*e*_	***Effective population size.*** An estimate of the number of breeding individuals in an ideal population as the actual population under consideration based on genetic variation [28]
Super-infection	Transmission (infection) by multiple mosquito bites, each bearing a unique, unrelated parasite genotype/strain [16]
Co-transmission	Transmission (infection) by a single mosquito bite bearing more than one parasite genotype/strain [16]

### Processing 24-SNP data

The 16S Bioinformatics with B4screening pathway, developed in a previous study [13], was used to clean the Miseq data for consistent frequency calls at each SNP site across 12 chromosomes. Briefly, the B4Screening pathway involves four steps: trimming adaptors from read sequences; sending them through the dada2 quality control pipeline for filtering, trimming, error rate detection, pairing, and chimera removal; removing mismatched primers; and finally, identifying amplicon sequence variants (ASVs) using a Random Forest Classifier. Following the above pipeline, samples with at least 16 SNP sites with a read depth of at least 500 were retained for SNP frequency determination and strain reconstruction [13].

After the above data preprocessing step, 360 out of 372 samples were included, comprising 65 samples from 1996, 71 samples from 2001, 48 samples from 2007, 77 samples from 2012, and 99 samples from 2017 for further data analysis.

### Data analysis

The statistical methods and metrics used to analyse and compare malaria strains, samples, and populations are as follows (see the Supplementary Information for more details).

### StrainRecon for constituent strains and STIM for MOI estimation

StrainRecon is an algorithm for disambiguating multiple strains in DNA samples using *n*-SNP barcodes. It estimates the strains in a sample for a given number of strains* K*. As input, StrainRecon takes a SNP frequency vector of length *n*, a proposed *K*, and, optionally, a noise level. The output consists of a maximum-likelihood estimate of the constituent *K* barcodes in the sample, their relative proportions, the goodness-of fit of this estimate, and an uncertainty estimation of each barcode [32]. STIM is an algorithm that uses StrainRecon to estimate the most likely MOI of a sample given its SNP frequency vector. Internally, STIM runs StrainRecon for different values of *K* and returns the *K* that best meets a defined threshold parameter for StrainRecon’s goodness of fit. This study used StrainRecon and STIM in conjunction with its 24-SNP barcode methodology to estimate both the constituent strains and MOI of each sample. To counteract potential noise in sample measurements and analysis methods, this study used only strains above the 5% proportion of total strains detected as a cutoff, following the methods of a previous study (details in Supplementary Information Method S1) [13].

### Analysis of MOI

Temporal trends in the MOI were tested for statistically significant differentiation. First, differentiation between any of the multiple distributions was evaluated using a Kruskal–Wallis test, and then differentiation between individual distributions was evaluated using Conover-Iman tests with False Discovery Rate (FDR) correction. In addition, the relationship between parasite density and MOI, age, and year of survey was analysed, both by year with FDR correction, and overall. Pearson’s correlation coefficient was first used to determine the correlation between variables, and univariate and multivariate linear regressions were used to determine the strength of these relationships. In particular, multivariate regression was used to assess the strength of the relationship between parasite density and MOI while controlling for age, a known confounding factor for parasite density in high-transmission areas. Further, a potential influence of age on MOI was examined by Conover-Iman test with Benjamini-Yekutieli FDR correction. Parasite density was log-transformed prior to statistical analysis. Pearson’s correlation and linear regression (both univariate and multivariate) calculations were performed using Python’s SciPy and statsmodels packages, respectively.

### Relatedness: FST and IBD

This study quantified the genetic relatedness of the parasite populations using *F*_*ST*_ and IBD. *F*_*ST*_ measures the relatedness of two populations by comparing the level of genetic differences between the populations relative to the level of genetic diversity within each population. The result is a value from [0,1]; values above 0.15 are considered significant differentiation, while values below 0.05 are considered non-significant differentiation, and values between 0.05 and 0.15 are considered moderate differentiation [23]. IBD measures the relatedness of individual strains by estimating the fraction of DNA segments inherited from a recent common ancestor, that is, the IBD-Fraction [25]. IBD-Fractions above 0.5 generally indicate significant relatedness.

This study used *F*_*ST*_ to compare the strain populations between years (details in Supplementary Information Method S2-1), and IBD to evaluate three measures (details in Supplementary Information Method S2-2): (1) within-year genetic relatedness (the relatedness of all combinations of strains in a single year), (2) across-year relatedness (the relatedness of all combinations of strains from two different years), and (3) within-host strain relatedness (the relatedness of all combinations of strains in a sample). For further validation, the results of (1) and (2) were compared with those of a baseline of randomly generated strains (details in Supplementary Information Method S3).

### Other population genetics metrics

Parasite strain populations were quantified using *H*_*s*_ and *N*_*e*_. The *H*_*s*_ metric is a variation of *H*_*e*_ that measures genetic diversity while accounting for sampling imbalances [38]. *N*_*e*_ is an indicator of the size of a population by estimating the number of breeding individuals in an idealized population [28]. Calculations of *N*_*e*_ were performed using both the Temporal and Linkage Disequilibrium methods. The details of the *H*_*e*_ and *N*_*e*_ analysis methods are provided in Supplementary Information Methods S5 and S6.

### Inferring superinfection and co-transmission

This study further analysed the correlation between within-host strain-relatedness and MOI to infer whether the complex infections were due to superinfection or co-transmission. Previous studies have shown that strains in superinfections tend to be less related than those in co-transmission [16, 17, 39]. Therefore, the relative changes in each transmission process over time can be inferred from changes in the correlation between subjects’ within-host strain relatedness IBD and MOI, measured using (1) Pearson coefficient and Pearson correlation regression line (i.e., the slope) and (2) Spearman’s monotonic correlation coefficient. The reason to use the two methods to measure correlations is because inferring superinfection and co-transmission from the relationship between MOI and within-host relatedness IBD is novel; using both methods allows us better interpretating results. The significance of these differences over time was quantified by 95% confidence intervals, generated using standard deviation calculations for Pearson’s slope and correlation coefficient and bootstrapping (n = 1000) for Spearman’s correlation coefficient. The p-values for Pearson and Spearman correlation tests were corrected using Benjamini-Yekutieli FDR correction. To further validate the changes in slope over time, analyses were conducted on two modified versions of the original dataset for modelling. The details are provided in Supplementary Information Method S4.

## Results

### Temporal trends of MOI

**Fig. 1:**
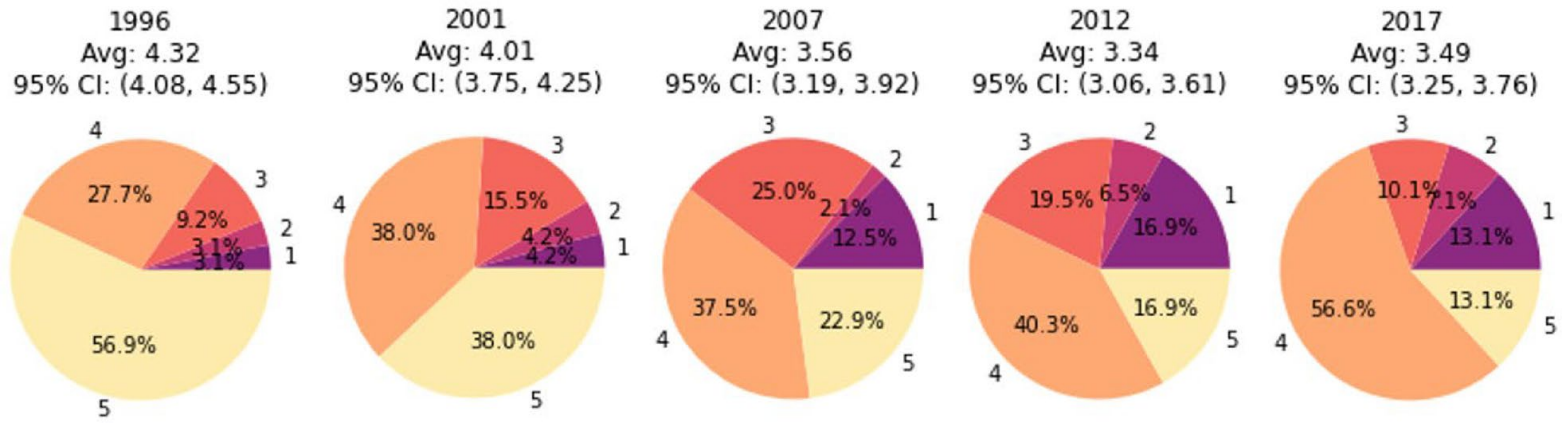
Mean MOI and prevalence of MOI by year. Pie charts show the proportions of samples with each MOI among the 65 samples from 1996, 71 samples from 2001, 48 samples from 2007, 77 samples from 2012, and 99 samples from 2017. The average MOI is listed above each pie chart, and confidence intervals were generated via bootstrapping (n = 1000)

The use of StrainRecon and STIM to reconstruct the strains in each sample revealed the trends in the MOI over time. Generally, the MOI steadily decreased from 1996 (average = 4.32) to 2012 (average = 3.34), with a slight increase in 2017 (average = 3.49). More specifically, from 1996 to 2012, the proportion of participants with five or more strains decreased (56.9–16.9%), and the proportion of participants with a single strain increased (3.1–16.9%). From 2012 to 2017, the proportion of samples with four strains increased (40.3–56.6%) (Fig. 1). These differences in MOI were statistically significant by a Kruskal–Wallis test for differentiation (*p* = 9.27e-8), as well as Conover-Iman tests [40, 41]. Conover-Iman tests showed that significant differentiation between the distributions in any two years except for 2001–2012, 2001–2017, and 2012–2017 (Fig. S1).

### Relationship between MOI and parasite density

A previous study conducted by this research group showed that parasite density ranging from 100 to 100,000/µl in mixed laboratory-cultured parasite strains did not influence the 24-SNP-based MOI estimates [13]. This study further examined the relationship between MOI and parasite density in a large set of field samples over time.

**Fig. 2:**
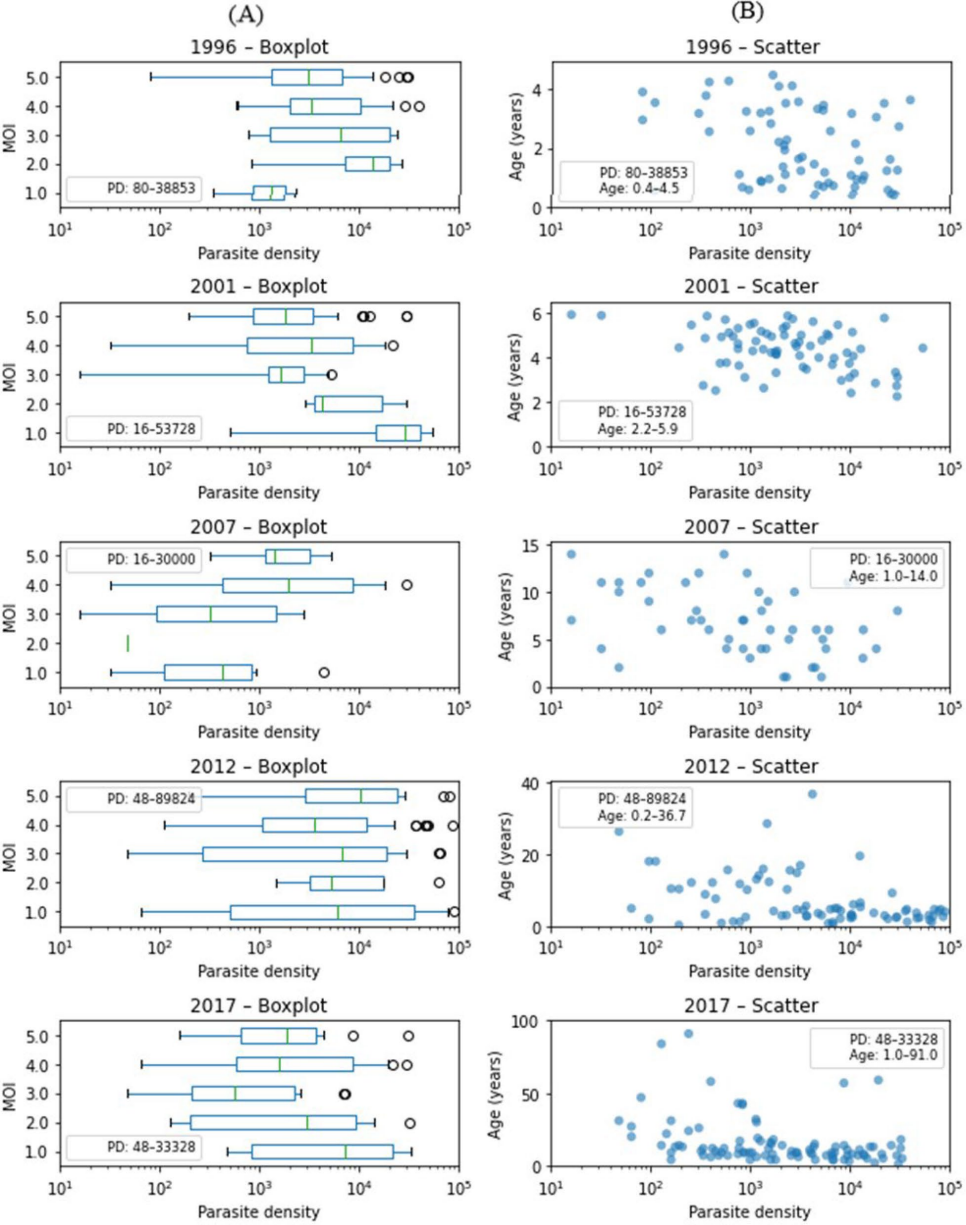

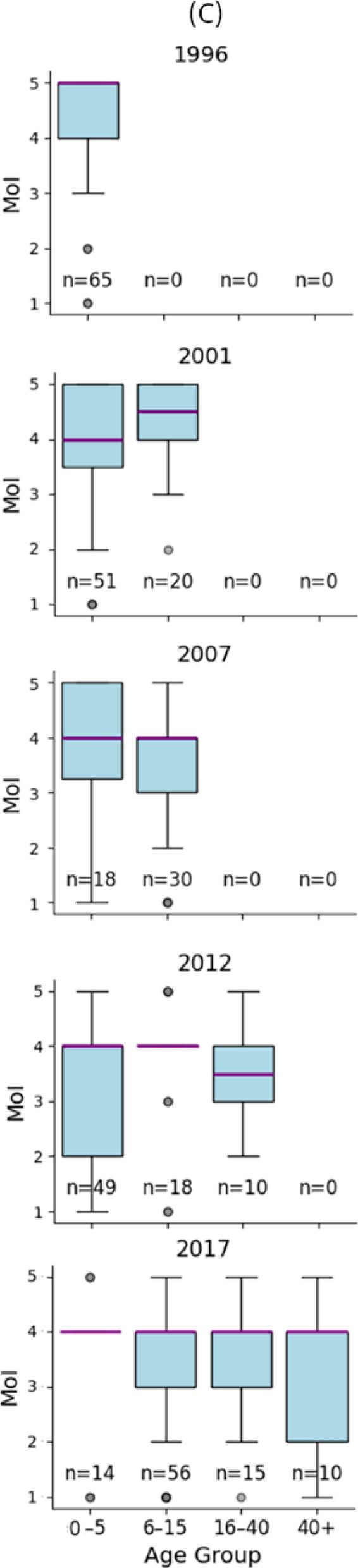
Plotting parasite density, age and MOI of each subject, stratified by year, to determine any confounding relationships. Two subjects from 2017 lacked parasite density data, and two subjects were missing age data, leaving 95 subjects in 2017. **A** Boxplots of parasite density versus MOI by year. Note that in 2007 only two subjects had MOI of 2, resulting in an incomplete boxplot. **B** Scatterplots of parasite density versus age by year. Parasite density ranges in **A** and **B** as well as age ranges in **B** are shown in the legends. **C** Boxplots of MOI versus age groups (0–5 years, 6–15 years, 16–40 years, > 40 years) by year. Numbers of subject (n) of each age group were shown in the legends

To visualize the range of variable values, boxplot of MOI and parasite density by year are shown in Fig. 2A and scatterplot of age and parasite density by year are shown in Fig. 2B. In Pearson correlation analyses, subjects’ parasite density showed no significant correlation with year (*p* = 0.20), no significant correlation with MOI (*p* = 0.671) and a significant (*p* < 0.001) inverse correlation with age (Table 2). In a multiple regression analysis including both MOI and age, no correlation was observed between MOI and parasite density. (R = −0.026, *p* = 0.736), while parasite density remained inversely correlated with age (R = −0.047 *p* < 0.001) (Table 3).

**Table 2:** Parasitaemia correlation analysis

Year	(A)			(B)		
	Age R	Age p-val	Age q-val	MOI R	MOI p-val	MOI q-val
1996	−0.32	0.009	0.02	−0.03	0.788	1.0
2001	−0.38	0.001	0.004	−0.15	0.207	1.0
2007	−0.42	0.003	0.009	0.37	0.010	0.112
2012	−0.40	0.000	0.004	0.06	0.607	1.0
2017	−0.34	0.001	0.004	−0.11	0.290	1.0
All	−0.27	0.000		0.02	0.671	

Pearson’s Correlation analysis was performed between log-transformed parasite density and (A) age and (B) MOI. Each analysis measured the relation (R), the *p*-value of the correlation (p-val), and the FDR-corrected *q*-value of the correlation (q-val) computed using the Benjamini & Yekutieli family-wise error rate correction method [51]

**Table 3:**
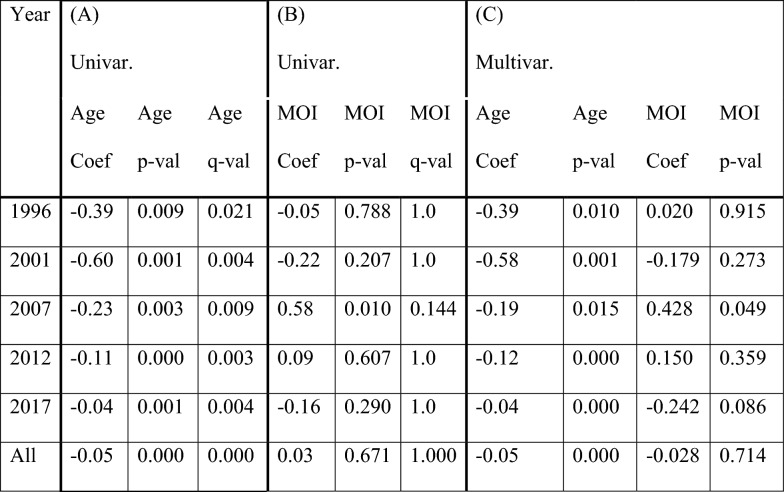
Association between parasite density, age, and MOI

Linear regression analysis was performed between log-transformed parasitaemia and (A) age using univariate regression, (B) MOI using univariate regression, and (C) MOI and Age using multivariate regression. Each regression measures the strength of the relationship (Coef), the *p*-value of the relationship (p-val), and the FDR-corrected *q*-value of the relationship (q-val) computed using the Benjamini & Yekutieli family-wise error rate correction method [51]

### Relationship between MOI and age

Because the ages of study subjects differ significantly in each survey year, especially 2007, 2012 and 2017 comprising older participants, a potential influence of age on MOI was further examined.

Boxplots of the age groups and the subjects’ MOI by year are shown in Fig. 2C. Conover-Iman test was used for comparing the MOIs between each age group, with Benjamini-Yekutieli FDR correction. Results showed no significant differences in MOIs between each age group in any year (Supplementary Information Fig. S2).

### Relatedness: FST and IBD

#### Distribution of different numbers of SNPs in strains within each year

**Fig. 3:**
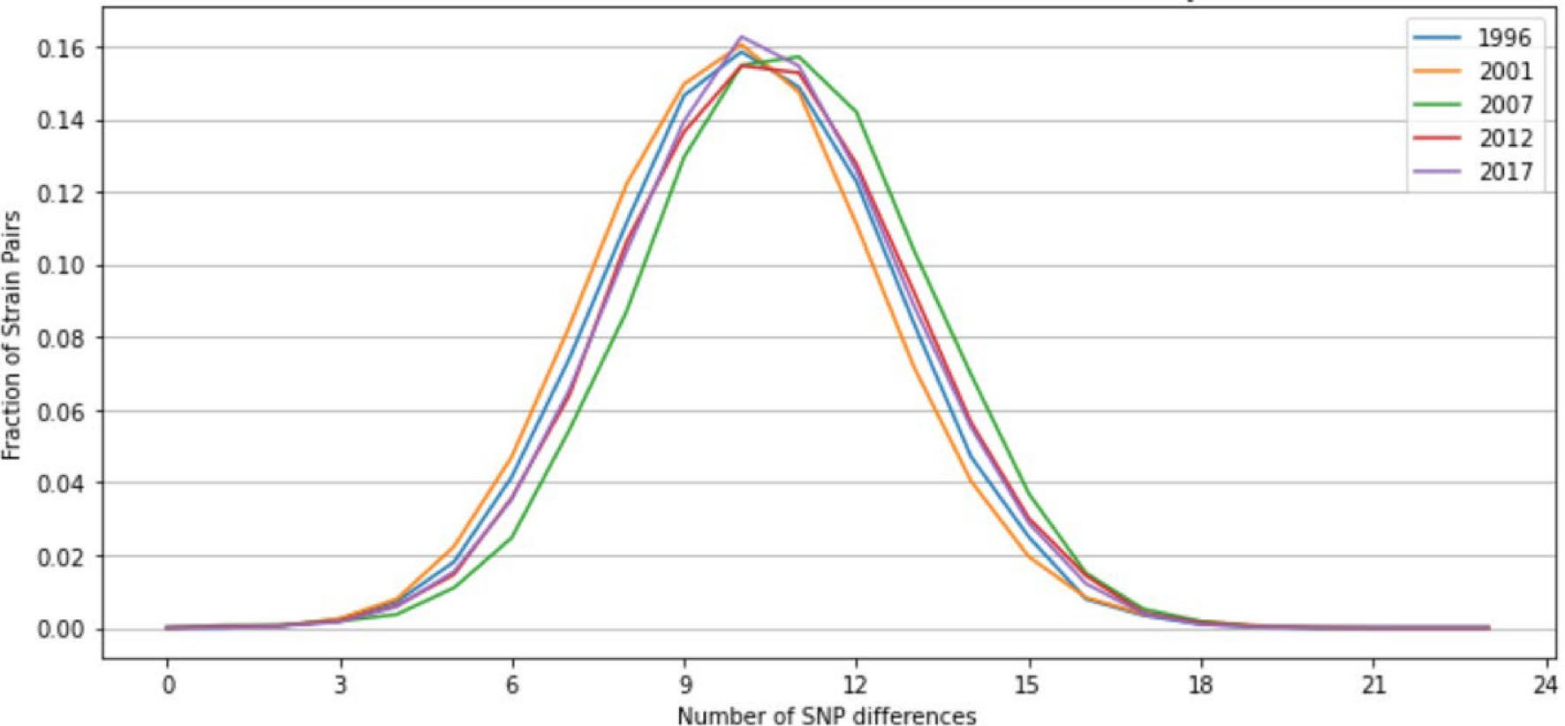
Distribution of different numbers of SNP differences in strains within each year. Strains within each year have a high number of SNP differences

Figure 3 presents the descriptive distributions of different numbers of SNPs in strains by year. It showed wide differences between strains, ranging from 3 to 18 SNP number differences with the average from 9 to 11 SNP differences in majority samples across the years. The simple descriptive results suggest that overall, the malaria parasite strains in Asembo area are highly polymorphic over time.

#### FST population relatedness

**Fig. 4:**
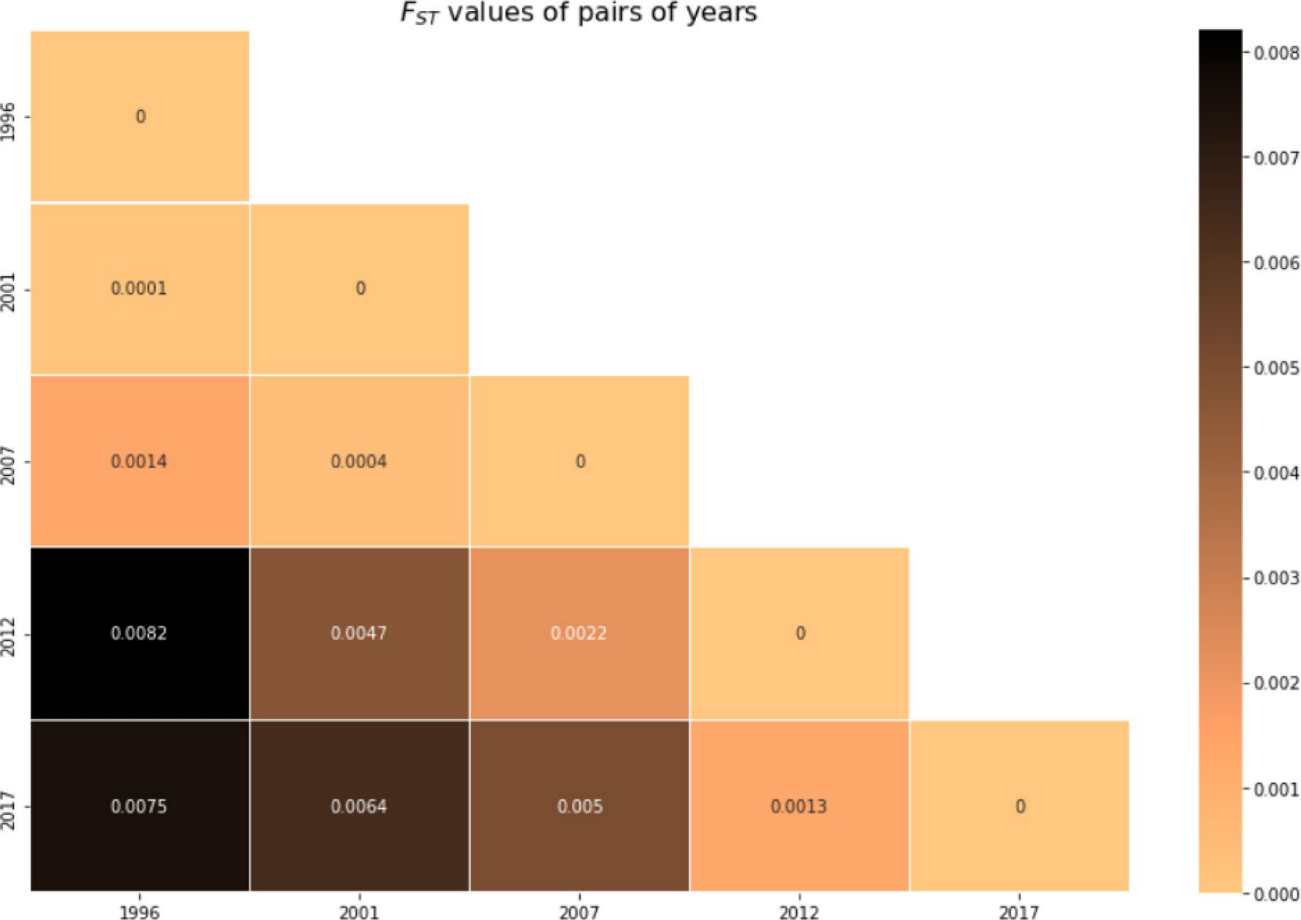
*F*_*ST*_ values between pairs of years. *F*_*ST*_ values indicate low (≤ 0.05) differentiation between years

The *F*_*ST*_ values between populations (Fig. 4) were negligible. Years closer temporally were more closely related genetically (with exception of 1996, which is slightly less related to 2012 than 2017), but the pairwise comparison of the most distinct pair of years (1996 and 2012, *F*_*ST*_ = 0.0082) was still below the 0.05 threshold associated with non-significant differentiation.

#### IBD strain relatedness within years and between years

**Fig. 5:**
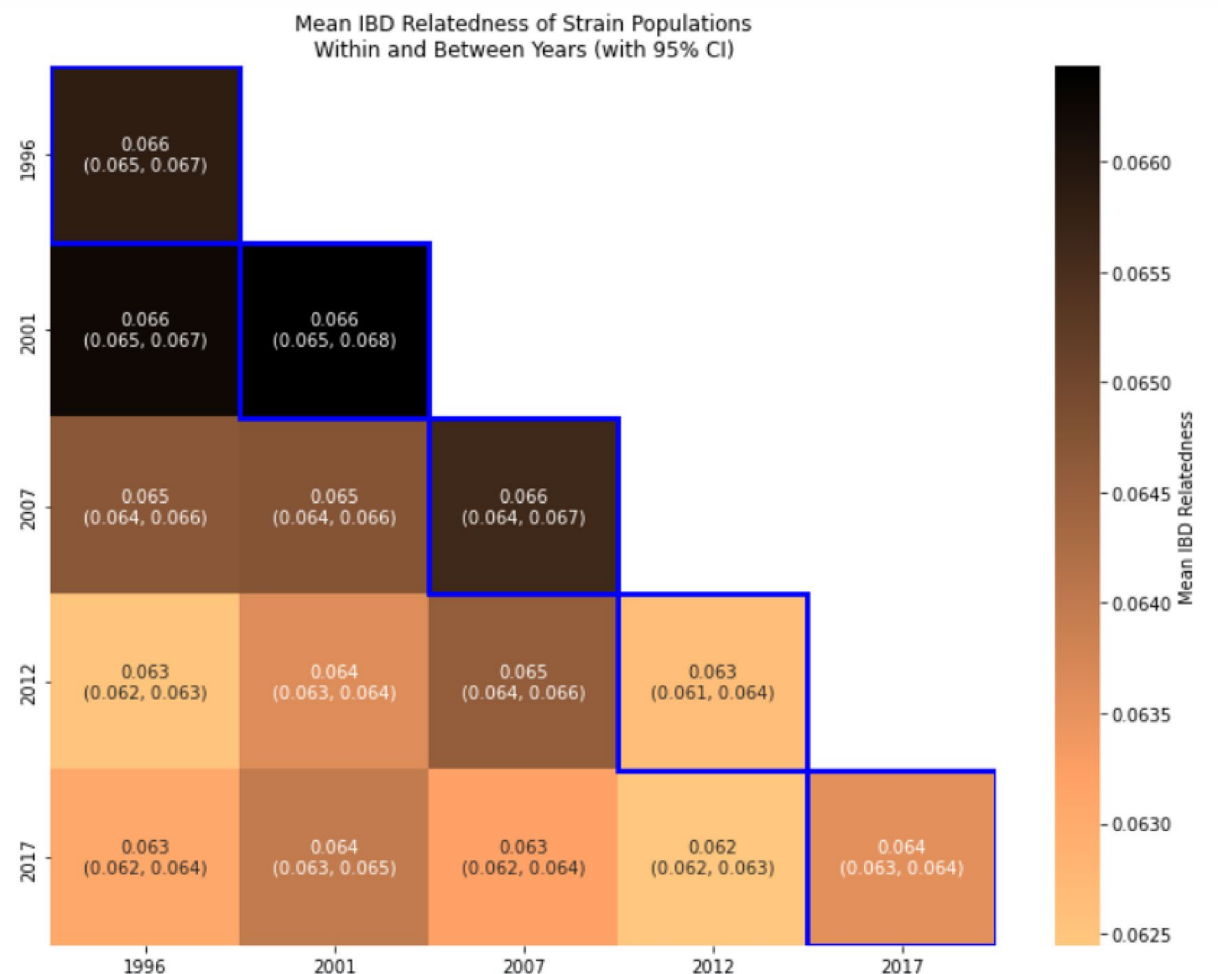
Mean values and 95% confidence intervals for strain relatedness values within-year and between-years over time. Confidence intervals were generated using bootstrapping with 1,000 resamples. Within-year values are given along the diagonal (highlighted in blue), whereas between-year values are given off-diagonal

Mean relatedness values were very low, as shown in Fig. 5. The highest mean relatedness values within years (0.066) and between years (0.065) were well below the threshold of statistically significant relatedness (0.5). Temporally, the mean relatedness values across years decreased from 1996 (0.066) to 2017 (0.064) based on changes in the 95% CIs over time. It also showed that strains between some years, such as between 1996 and 2001were more related than those between 2012 and 2017, based on 95% CIs. To rule out random chance in changes of the low IBD values, further comparison of the relatedness of these strains to those within a randomized baseline revealed no discernible difference (details in Supplementary Information Method S3 and Figs. S3-1, 3–2). Overall, these results suggest that from 1996 to 2017, the malaria parasite strain populations of western Kenya: (1) diversified within years, and (2) were highly diverse and well mixed across years.

#### IBD strain relatedness within hosts

**Fig. 6:**
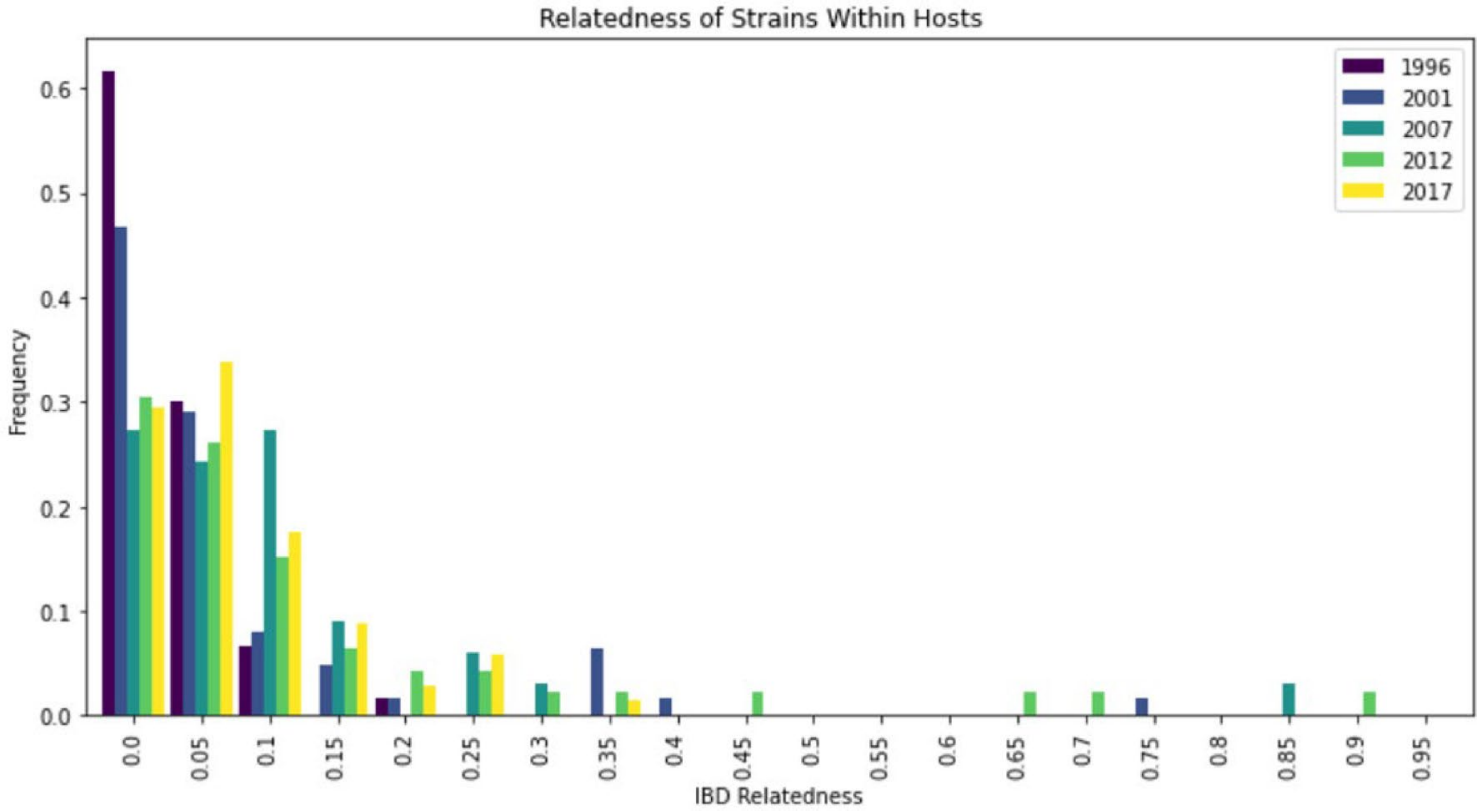
Within-host strain-relatedness histogram. The frequencies of individuals’ within-host strain-relatedness values from 1996 are shown in purple, 2001 in blue, 2007 in teal, 2012 in green, and 2017 in yellow. Only strains accounting for at least 5% of the total strains were analysed

**Fig. 7:**
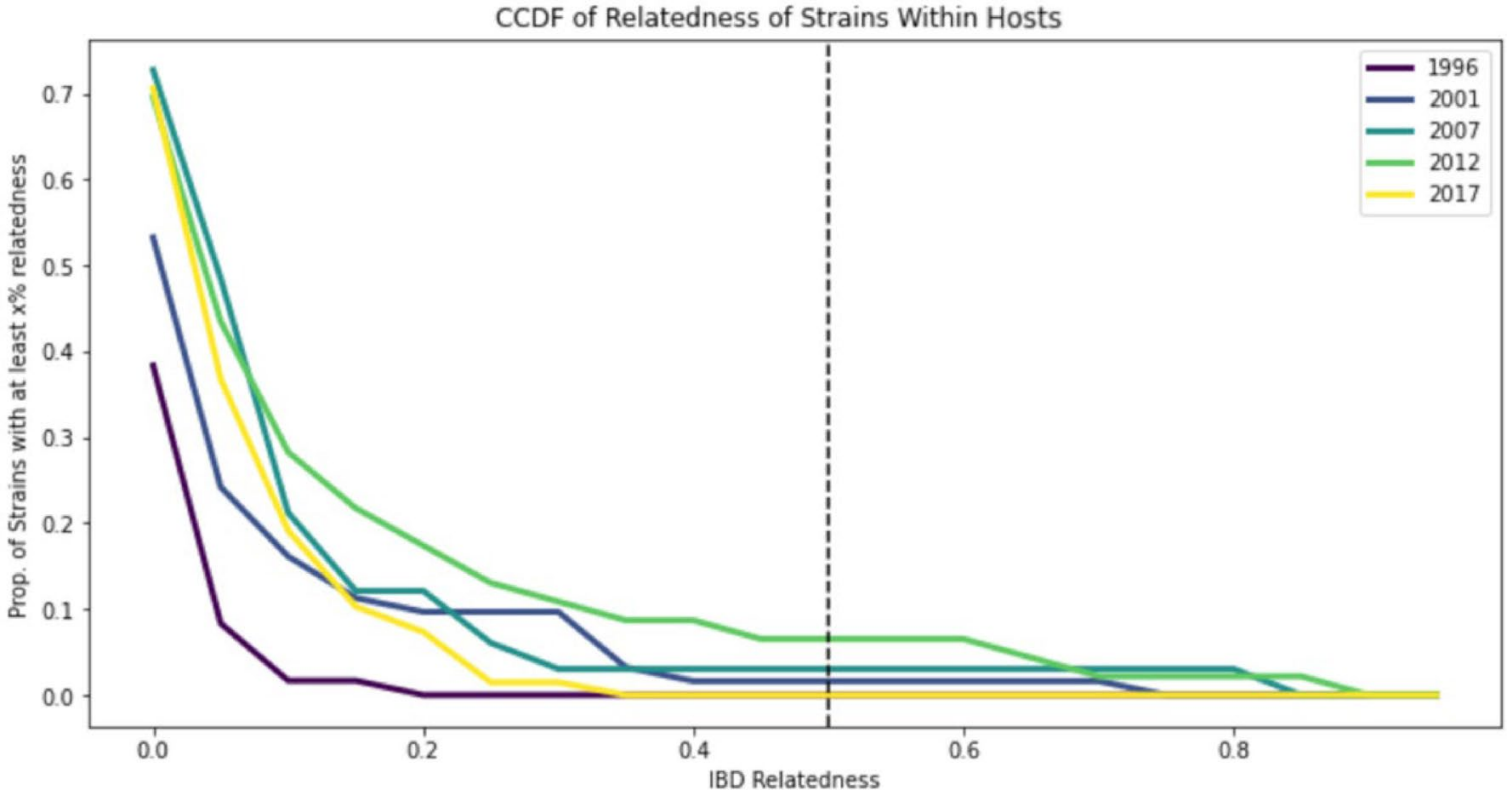
Complementary Cumulative Distribution Function (CCDF) of the within-host strain relatedness plot. The within-host strain-relatedness CCDF shows the proportion of strains that have *x*% or more relatedness. 1996 is shown in purple, 2001 in blue, 2007 in teal, 2012 in green, and 2017 in yellow. The commonly accepted threshold for relatedness (0.5) is indicated by a dashed line [24]

Overall, IBD calculations showed a heavy skew towards low strain-relatedness within-host (Fig. 6). This trend varied from its lowest in 1996 to its highest in 2012 (Fig. 7). Further analysis showed that even in the year with the highest IBD estimates (2012), only 6.5% of subjects had a within-host relatedness greater than 0.5 (Table 4). Broadly, strain-relatedness increased from 1996 (samples with IBD > 0.5 = 0.0%) to 2012 (samples with IBD > 0.5 = 6.5%), and then decreased again from 2012 to 2017 (samples with IBD > 0.5 = 0.0%) (Table 4). Conover-Iman tests with FDR correction indicate that the increase from 1996 to 2012 was statistically significant (p < 0.01), though the decrease from 2012 to 2017 was not (p = 0.49) (details in Supplementary Information Fig. S4). Overall, the results suggest that (1) most study participants were infected with multiple unrelated strains and (2) IBD-based strain-relatedness could be a sensitive metric for assessing temporal changes in within-host strain-relatedness in high transmission areas. Moreover, the significant increase in the percentage of samples with IBD > 0.5 from 1996 to 2012 and the decrease from 2012 to 2017 warranted further investigation of the relationship between within-host strain relatedness (IBD value) and MOI (number of strains).

**Table 4:** Percentage of subjects, by year, with within-host IBD strain relatedness above 0.3, 0.4, and 0.5

Year	IBD > 0.3 (%)	IBD > 0.4 (%)	IBD > 0.5 (%)
1996	0.0	0.0	0.0
2001	9.7	1.6	1.6
2007	3.0	3.0	3.0
2012	10.9	8.7	6.5
2017	1.5	0.0	0.0

### H_s_ and N_e_

#### Expected heterozygosity (H_s_)

**Fig. 8:**
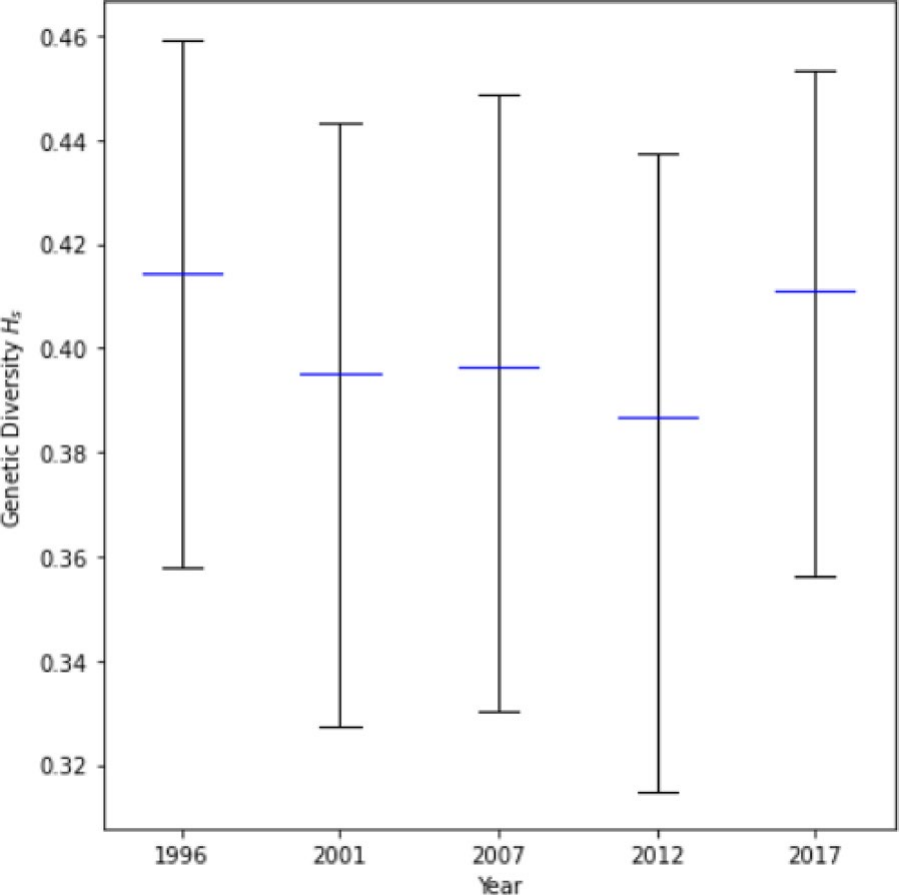
Genetic diversity over time, as measured by *H*_*s*_. The blue midlines show the mean value for each year, while the error bars represent the 95% confidence intervals

*H*_*s*_ values of each year’s reconstructed strains were high, ranging from 0.38 to 0.41, as shown in Fig. 8. Mean values decreased from 1996 (mean = 0.41) to 2012 (mean = 0.38) before increasing again in 2017 (mean = 0.41); however, the confidence intervals overlapped for all years. Overall, *H*_*s*_ calculations indicated that the parasite strains in Asembo were indistinguishably diverse at the population level over time.

#### Effective strain population size (N_e_)

**Fig. 9:**
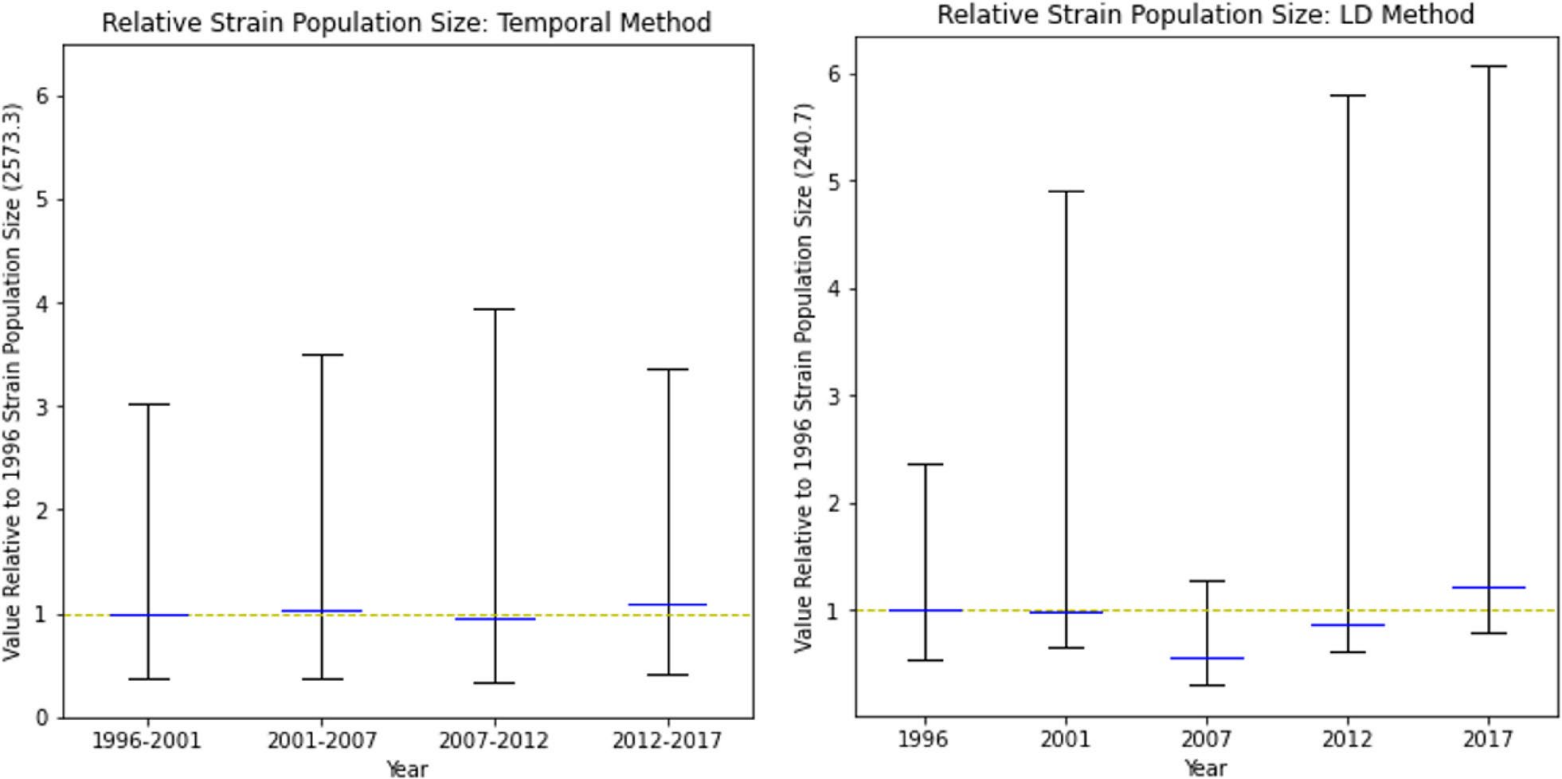
Effective population size estimates. Values shown are relative to those of 1996. A horizontal yellow line at y = 1 was added for ease of comparison. Calculations were performed using **a** the Temporal method on the four pairs of consecutive years and **b** the Linkage Disequilibrium (LD) method for each of the five years. Blue midlines indicate the estimated relative value of *N*_*e*_, and error bars indicate 95% confidence intervals. Initial *N*_*e*_ estimates for 1996 are shown in parentheses on the Y-axis. Normalisation to the 1996 estimates allowed comparisons between the two methods (exact *N*_*e*_ numbers are provided in the Supplementary Information Table S1)

The confidence intervals of the estimated effective population size for the years have a substantial width, overlapping with all other years, as shown in Fig. 9. Consequently, no significant temporal differentiation in the effective strain population size was observed.

### Inferring superinfection and co-transmission from within-host IBD and MOI

**Fig. 10:**
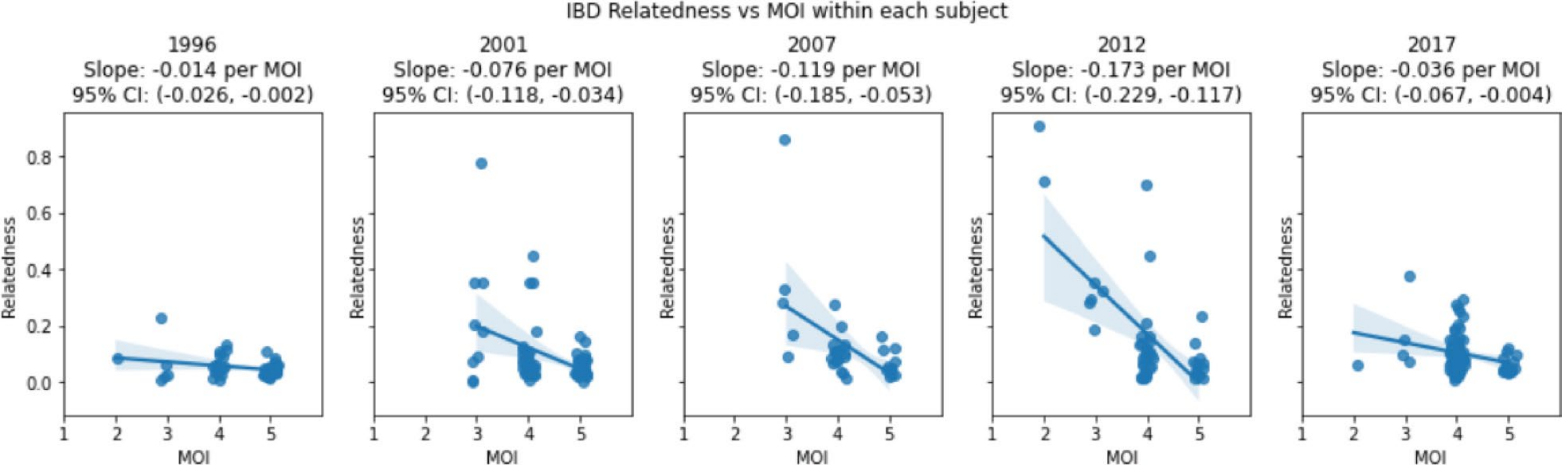
Relationship between subject’s with-host strain relatedness and their MOI. The analyses of the relationships were separated by year. Because the MOI values are discrete, jitter was added along the MOI axis to avoid overplotting. The slope of the correlation and the 95% confidence interval for the slope are given above each plot

As the 24-SNP-based MOI and IBD-based within-host strain relatedness are both metrics at the individual level, there is a potential utility in using the relationship between the two metrics as a proxy (slope) for inferring the transmission process. Correlation analyses suggest a statistically significant, inverse, monotonic relationship between MOI and within-host IBD relatedness (Table 5). Spearman’s ρ-test showed statistically significant, monotonically inverse correlations in the years 2001, 2007, 2012, and 2017 (q ≤ 0.05), but was not significant in 1996 (q = 0.287). Pearson’s correlations further showed statistically significant inverse linear correlations for all years (q ≤ 0.065), particularly during the 2001–2012 period (q ≤ 0.005) (Table 5). Both Pearson’s and Spearman’s correlation coefficients suggest an increase in inverse correlations between 2001 and 2012, followed by a decrease in 2017. To further visualize the change in the inverse correlation between the subjects’ within-host relatedness IBD and MOI over time, the slopes are presented in Fig. 10, the data of which were also presented in Table 5. The correlation was weakly negative in 1996 (slope = −0.014) and became increasingly negative until 2012 (slope = −0.173), before rebounding in 2017 (slope = −0.036). The 95% confidence intervals indicate that these differences are significant between the Pearson’s slope values of 1996 vs 2007, 1996 vs 2012, and 2012 vs 2017 (p < 0.05) (Fig. 10 and Table 5). To verify the trends of the observed changes in slope over time, further analyses were conducted on a baseline model (in which strains were randomly shuffled between subjects) and a high-proportion model (in which only higher-proportion strains were considered in subjects) (the details in Supplementary Information Method S4). The baseline model exhibited none of the inverse correlations seen in the actual data while the higher-proportion-strain analysis models showed similar inverse trends (slopes) of correlations (the details in Supplementary Information Figs S5-1, 5–2, 5–3). These results suggest that the inverse trends and changes existed in the real data that are not simply artefacts of the analysis process, and the overall significance of these changes in slopes was further confirmed using the baseline model and higher proportion models. Because the relatedness of strains from superinfections tends to be relatively less than that of strains from co-transmissions, these slope trends suggest that (1) the transmission process increased towards co-transmission between 2001 and 2012, and then (2) towards superinfection in 2017 in Asembo, western Kenya.

**Table 5:**
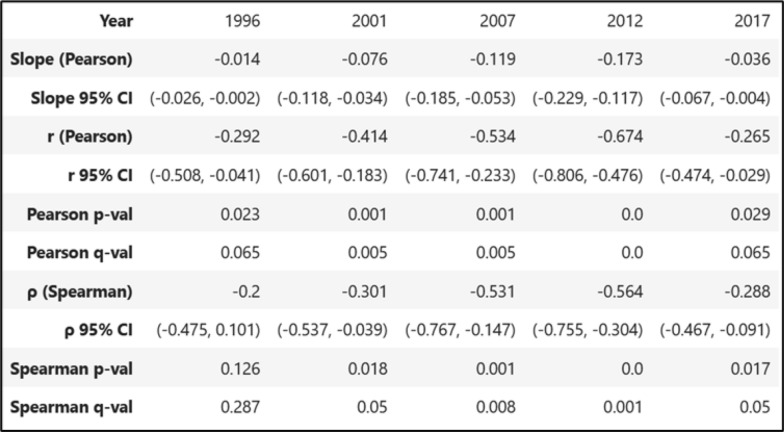
Correlation of within-host relatedness IBD and MOI of each subject across each year

The table shows the results of statistical tests for both Pearson and Spearman correlation. The r (Pearson) coefficient is the strength of their linear correlation while the ρ (Spearman) coefficient shows the strength of their monotonic (ranked) correlation. Pearson’s test gives the slope linear correlation regression line between the variables. The p-values quantify the likelihood of non-zero correlations, while q-values adjust false discovery rate (FDR) of the p-values

## Discussion

This longitudinal parasite genomic analysis using within-host metrics revealed distinct temporal patterns in malaria transmission dynamics in Asembo, western Kenya, from 1996 to 2017. Most notably, a steady decline in the mean MOI from 4.32 to 3.34 between 1996 and 2012, followed by a slight resurgence to 3.49 in 2017 was observed. This resurgence coincided with a shift in transmission mechanisms: the novel slop metric indicated that co-transmission increased during the period of declining MOI (2001–2012), whereas superinfection returned to predominance in 2017 as in 1996. These findings show that changes in MOI reflect not only parasite diversity but also fundamental shifts in transmission processes. In this study, there was no significant differentiation in* F*_*ST*_, *H*_*s*_, *N*_*e*_, or strain-relatedness at the population level over time. Overall, the results demonstrate that detecting temporal changes in malaria transmission in high-transmission settings requires analytical methods tailored to high-diversity parasite populations for understanding the changes in transmission dynamics.

**Fig. 11:**
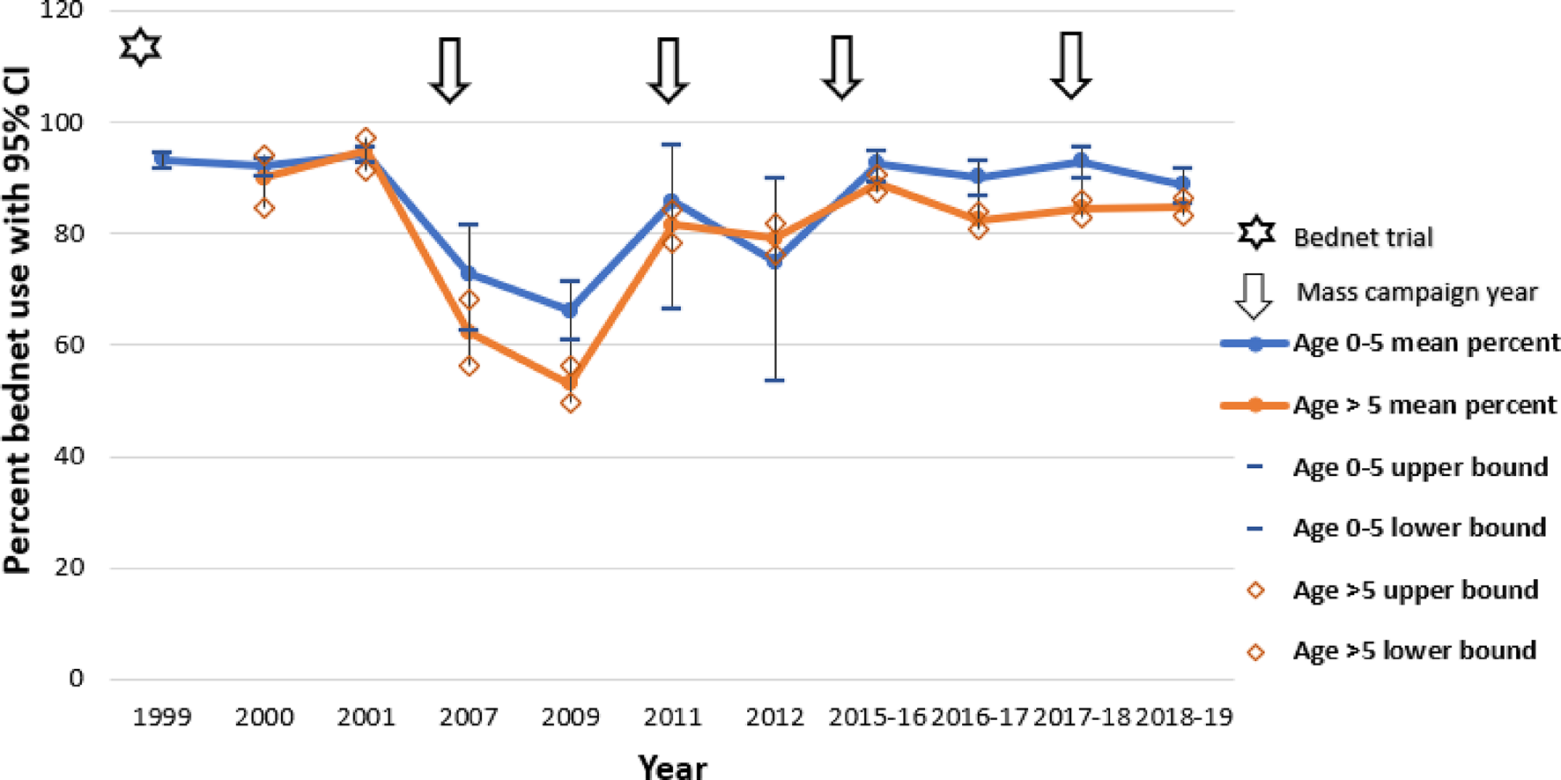
Bed net intervention data from 1996 to 2019 in Asembo, Siaya, western Kenya. Lines indicate bed net usage defined as the proportion of people that reported sleeping under a net the previous night. Stars and arrows indicate the large-scale bed net trial (1996–2001) and bed net distribution campaigns 2006, 2011, 2014 and 2017/2018. The 2017/2018 campaign did not occur until after the collection of samples in this study

By leveraging StrainRecon and STIM for NGS-24-SNP-based MOI estimation, this study showed that the mean MOI steadily decreased from 1996 (average = 4.32) to 2012 (average = 3.34), with a slight increase in 2017 (average = 3.49). A similar trend was also reflected by a decrease in the proportion of participants with five or more strains (56.9% to 16.9%) and an increase in the proportion of participants with a single strain (3.1% to 16.9%) from 1996 to 2012. From 2012 to 2017, the proportion of samples with four strains increased (40.3% to 56.6%). Overall, this suggests a continuous reduction in transmission intensity from 1996 to 2012 and a slight resurgence between 2012 and 2017 in the Asembo area. To assess the potential contributions to the MOI increase in 2017, bednet campaign and bednet usage were examined (Fig. 11). The trend of bednet usage over time does not track the MOI trend. However, sample collection in 2007 and 2012 occurred one year after the mass bednet campaigns (2006 and 2011, respectively), while the sample collection in 2017 was done after the 2014 bednet campaign but before the bednet campaign conducted at the end of 2017 and early 2018. Taken together and accounting for a likely time lag between bednet campaigns, this suggests that the NGS-24 SNP-based MOI estimation using the StrainRecon and STIM analytical tools is a sensitive metric for temporal genomic surveillance of *P. falciparum* in Asembo, western Kenya, and the temporal change in the 24-SNP-based MOI can be used as a metric for evaluating the impact of bednet coverage on transmission intensity. This is further supported by the previous study of NGS-based 24 SNP method development, showing the decrease in 24-SNP-based MOIs and proportions of samples with 5 strains from 1996 to 2012 are in tandem with the decline in EIR and malaria prevalence during the same time period [13]. Turning points, however, differ with a sharp decline in EIR and the stagnation of malaria prevalence from 2007 to 2012, suggesting a non-linear scaling relationship among the MOI, EIR and prevalence [13].

Unlike the within-host MOI metric, temporal analysis of other traditional genetic metrics at the population level using the same 24-SNP dataset was largely uninformative. There was no strain-relatedness at the population level using IBD analysis within and between years (Fig. 5). The analysis also showed no significant differentiation in *F*_*ST*_*, H*_*s*_*,* and *N*_*e*_ at the population level over time (Figs. 4, 8, and 9). The lack of power for these population genetic metrics in detecting parasite strain population dynamics in Asembo, a high transmission area, could be attributed to several factors. The use of 24-SNP sites in this study might have been too sparse for some traditional population genetic metrics. Previous studies corroborate this, suggesting that 24-SNPs are inadequate to accurately capture IBD-based parasite dynamics at the population level and that 200 or more SNPs may be required [24, 42]. Since the malaria parasite population in western Kenya is abundant, diverse, and well-mixed at the population level (Fig. 3 and Supplementary Information Fig. S4), a high recombination rate is expected, which would result in low values of IBD and insignificant *F*_*ST*_ [23, 25]. It is also possible that the substantial width in 95% CIs, overlapping with all years, for *N*_*e*_ estimate may stem from latent uncontrolled factors in the sampling of the underlying population, such as a locally high genetic diversity background as a main factor as well as inadequate sample size, which might limit the usefulness of this specific metric for inferring transmission intensity when the infection rate is high [28].

By contrast, the within-host strain-relatedness analysis using IBD provided insights into the parasite strain dynamics. Overall, a heavy skew towards low within-host strain-relatedness was observed over time (Fig. 6), varying from low relatedness in 1996 to relatively high relatedness in 2012 (Fig. 7). Further analysis showed that the IBD strain relatedness gradually increased from 1996 (subjects with IBD > 0.5 = 0.0%) to 2012 (subjects with IBD > 0.5 = 6.5%), and then decreased again from 2012 to 2017 (subjects with IBD > 0.5 = 0.0%) (Table 4). The results suggest that although most study participants in Asembo were infected with multiple unrelated strains, the IBD-based estimate is a sensitive metric for assessing within-host strain relatedness in a high transmission area. The trend of change in within-host strain-relatedness from 1996 to 2017 (Table 4) accords with the trend observed in MOI over time (Fig. 1). Moreover, the within-host IBD-based estimations are made on a per-sample basis and thus may have the potential for spatial clustering analysis to identify transmission hot spots in high transmission areas. Especially, repeated sampling for evaluation of changes in the within-host IBD and MOI over the course of infection in short timescale may shed light on transmission dynamics of malaria hot spots at a finer resolution in high transmission areas. Previous studies have shown that genotype profiles based on polymorphism of surface antigen genes in high transmission settings can fluctuate substantially over the course of an infection [43, 44]. Further investigation of such utility by 24- SNP-based within-host IBD as well as MOI is needed. In addition, the impact of transmission intensity, particularly in high transmission settings, on the spread of antimalarial drug resistance remains controversial. This is partly due to the difficulty of measuring within-host competition between different parasite clones or strains, among other confounding factors [45]. The use of within-host IBD metrics may offer an indirect means of assessing strain competition based on the assumption of less competition when co-infecting strains are more closely related than when they are unrelated. This requires further investigations too. Overall, while strain-relatedness-based metrics at the population level are subject to the number of SNPs used, the within-host metrics MOI and strain-relatedness IBD based on 24 SNPs are robust in Asembo, a high transmission area.

This study further explored the relationship between the 24 SNP-based MOI and IBD-based within-host strain-relatedness at the individual level. As strains in superinfections tend to be less related than those in co-transmissions [16], the change in the relationship between the two within-host metrics (slopes) over time provides a quantitative method for inferring temporal changes in transmission dynamics. This study found a consistent inverse correlation linearly and monotonically between within-host relatedness IBD and MOI using Pearson’s and Spearman’s coefficient tests. The coefficients from both tests suggested an increase in inverse correlations between 2001 and 2012, followed by a decrease in 2017 (Table 5). The visual slope of this relationship varied significantly over time, being weakly negative in 1996 (slope = −0.014), becoming increasingly negative until 2012 (slope = −0.173), and then rebounding towards less negative in 2017 (slope = −0.036) (Fig. 10 and Table 5). The overall significance of these temporal changes in slopes was further verified using a baseline model and a high-proportion model (Supplementary Information Figs. S5-1, S5-2, S5-3). These slope trends suggest that the transmission process shifted towards co-transmission from 2001 to 2012, before superinfection returned to predominance in 2017, resembling patterns observed in 1996 in Asembo. This finding provides a mechanistic explanation for the slight resurgence in the MOI in 2017 (Fig. 1), demonstrating that changes in the MOI reflect not only fluctuations in within-host parasite diversity but also shifts in the underlying transmission mechanisms. The findings from this study are supported by a previous transmission modeling study, suggesting the contribution of co-transmission and superinfection to genetic diversity shifts as a function of transmission intensity [6].

This within-host slope-based approach developed in this study represents an analytical methodological advancement over previous studies. A recent study conducted in a low transmission area of Senegal relied on within-host heterozygosity measurements (Rh with threshold) via 24-SNP barcode data to infer co-transmission vs. superinfection [17], but this approach was not designed for temporal analysis in high-transmission settings. By leveraging the individual strain estimates of StrainRecon, this is the first study to use a simple within-host slope metric (the relationship between within-host strain relatedness (IBD) and MOI to infer temporal changes in transmission processes in Asembo, a high transmission area.

While acknowledging that parasite prevalence surveys and entomological inoculation rates remain the cornerstone metrics for malaria control programs, the slope metric offers complementary insights that may prove valuable in specific contexts. For instance, when the MOI declines following intervention implementation, traditional surveillance cannot distinguish whether this reflects reduced mosquito-human contact (successful vector control) or changes in mosquito infection patterns (shifts in the parasite population structure). The temporal change in slope metric addresses this analytical gap by providing mechanistic insights into transmission dynamics that align with recent calls for more nuanced genomic surveillance approaches in diverse epidemiological settings [46].

Further validation of this slope metric across diverse high-transmission settings and a systematic evaluation of its utility in programmatic decision-making are necessary to determine its broader applicability for malaria surveillance. Future studies should also explore the relationship between the within-host genetic metrics and traditional epidemiological measures, such as parasite prevalence and EIR, as recommended for validating novel surveillance approaches [4]. However, the demonstrated ability to detect temporal shifts in transmission mechanisms using routinely collected smear-positive samples for NGS- based-24 SNP strain barcode test suggests that this approach could enhance our understanding of the impact of interventions on parasite population dynamics.

A previous study on NGS-based 24-SNP method development conducted by this research group showed that parasite density did not influence the SNP frequency calls used for MOI estimates in mixed laboratory-cultured parasite strains (parasite densities ranging 100–100,000/µl) using the novel bioinformatics pipeline [13]. The current study further examined the relationship between parasite density and 24-SNP-based MOI estimated by the same StrainRecon and STIM tools in a large set of smear-positive field samples over time to test whether there was a confounding influence of parasite density on the MOI estimation. Indeed, the present study verified that there was no correlation between 24-SNP-based StrainRecon and STIM MOI estimations and the parasite densities in the field samples collected across multiple years (Fig. 2A, B, and Tables 2 and 3), removing the need for statistical control for 24SNP-based MOI estimations in field samples as well as temporal analysis of the 24-SNP-based MOI when parasite densities varied across years. In addition, the verified results from field samples warrant the potential utility of StrainRecon with the NGS-based 24-SNP barcode laboratory tools and newly advanced unique algorithms (manuscript under preparation) for distinguishing recrudescence from new infection in antimalarial therapeutic efficacy trials. Lastly, current study further showed no significant differences in MOIs between age groups at each survey year, suggesting no effect of ages on 24 SNP barcode-based MOI over time. The results are supported by previous studies [13, 47]. Together, this shows that the temporal changes in the MOI and the negative slope metrics observed in this study reflect the transmission dynamics over time. The 24-SNP-based MOI estimations not influenced by ages could be explained by the nature of neutral and unlinked 24 SNP targets across 12 of 14 chromosomes within *P. falciparum* genome unlikely under host immune selection.

The limitation of this study is that routinely collected blood smear-positive field samples were used for analysis. The reason for only employing the smear-positive samples for current study was based on the findings from the previous NGS-based 24SNP method development [13]. The NGS-based 24SNP method, unique bioinformatics and StrainRecon and STIM algorithms substantially improved the sensitivity and accuracy in detection of minor strains (haplotypes) in parasite densities within microscopic detection ranges but accuracy in detection of minor strains in low densities of parasite decreased. Many studies using more sensitive molecular methods (i.e., PCR) in research contexts have shown that infections with parasite densities below detection threshold by routine microscopic test (submicroscopic infections) are present across a range of different areas of low and high transmission intensity including the current study area, and such submicroscopic infections potentially contribute to transmission [48, 49]. Therefore, the results from current study could not be generalized at population level and it remains unknown for transmission dynamics in submicroscopic parasite population in the study area. Nevertheless, the within-host strain analyses and the metrics explored by current study are valuable for conventional malaria surveillance that is the core of malaria programs in high transmission areas.

## Conclusion

This study shows that malaria genomic surveillance in high-transmission setting requires fundamentally different approaches from those developed for low-transmission area. While traditional population-based genomics metrics proved uninformative in the highly diverse parasite populations in this study, the within-host-based strain analyses, particularly the novel slope metric, revealed subtle but important shifts in transmission dynamics that would be invisible to conventional surveillance. The ability to distinguish between superinfection and co-transmission in smear- positive parasite population using the simple slope (correlation between within-host IBD and MOI) that is built upon NGS-based 24-SNP barcode combined with StrainRecon and STIM algorithms for within-host strain reconstruction allows us to assess whether observed changes in MOI reflect successful reduction in mosquito-human contact or merely shifts in parasite population structure. Further validation of the NGS-based within-host IBD, MOI and slope metrics across diverse high-transmission settings and evaluation of their utility are necessary to determine applicability for malaria genomic surveillance.

## Supplementary Information

Supplementary material 1.

Supplementary material 2. Table S1: Effective population sizes for all years.

Supplementary material 3. Fig. S1 Comparison of MOI between years. Conover-Imam *q*-values are given of pairwise comparisons across years.

Supplementary material 4. Fig. S2 Comparison of MOI between age groups by year. Conover-Iman test was used for comparing the MOIs between each age group, with Benjamini-Yekutieli FDR correction. Note that 1996 was excluded for analysis because all subjects in that year were in one group <=5 years old. The q-values indicate no significant differences in the MOIs between each age group in each year.

Supplementary material 5. Fig. S3 1 IBD strain-relatedness compared to baseline. CDF of frequencies of IBD strain relatedness are shown within years on the diagonal and across years off the diagonal. Each pair was compared against its baseline pair, where each year’s baseline was made from strains randomly drawn from the distribution of SNPs. 2 KS tests comparing the relatedness of strains within and across years to their baselines. The *p*-values of these comparisons (left), as well as the *q*-values obtained by performing two-stage non-negative false discovery rate corrections (right), do not exhibit statistically significant differences between the real distributions and their baselines.

Supplementary material 6. Fig. S4 Conover-Iman statistical q-values with FDR for differentiation of within-host relatedness between years. Lower values indicate more significant differentiation between years, and higher values indicate less significant differentiation. 1996 and 2001 show greater differentiation from other years than 2007, 2012, and 2017.

Supplementary material 7. Fig. S5 1 Analysis of the relationship between baseline within-host relatedness and MOI with strains shuffled across subjects within each year. Slopes of MOI vs within-host relatedness show none of the trends seen in empirical data, suggesting that the trends found are a result of true patterns in the samples analysed and are not an artefact of the analysis itself. 2 Analysis of the relationship between within-host relatedness and MOI in high-proportion (>=10%) strains. The result of analysing only strains exceeding 10% proportions is shown. Results reflect the trends seen in empirical data, suggesting that the trends found are a result of true patterns in the samples analysed and are not an artefact of the analysis itself. 3 Analyses of the relationship between within-host relatedness and MOI in high-proportion (>=20%) strains. The results of analysing only strains exceeding 20% proportions are shown. Results reflect the trends seen in empirical data, suggesting that the trends found are a result of true patterns in the samples analysed and are not an artefact of the analysis itself.

## Data Availability

The source code for the B4Screening pathway for bioinformatics pipeline and STIM analysis tool is available online at https://www.ymsir.com/stim/. All Sequence Read Archive (SRA) data for this study were submitted to the NCBI BioProject under accession no. PRJNA 555848 for the Kenya samples from 1996, 2001, 2007, and 2012 [13]. And no. PRJNA1229632 for the Kenya 2017 samples, available online at https://www.ncbi.nlm.nih.gov/sra.
