# Supplementary material for: Assessing the impact of temporal changes in transmission on Plasmodium falciparum strains in Asembo, western Kenya (1996–2017) using within-host metrics via 24-SNP barcodes
Source: Malar J. 2025 Dec 17;25:49. doi: 10.1186/s12936-025-05700-3 (PMC12829202; doi:10.1186/s12936-025-05700-3)
Supplement: Supplementary file 1 — Supplementary material 1. [file 12936_2025_5700_MOESM1_ESM.docx]

## **Supplementary Information**

### **Method S1: StrainRecon and STIM**

StrainRecon estimates the strains in a sample by treating the problem as a mathematical inverse optimization problem, using maximum-likelihood estimation to solve for the matrix **M** and vector *w* in the equation

$$\boldsymbol{M}w = d$$

for a given number of strains *k,* where *d* is a vector over $\left[ 0,1 \right]^{n}$ representing the measurements at each of the *n* SNP sites, ***M*** is the binary matrix representing the strains in the sample, and *w* is a vector representing the proportions of each strain in the sample. Meanwhile, the STIM algorithm is used to predict the most likely value of *k,* estimating matrix **M** and vector *w* for various *k* and selecting *k* based on the goodness-of-fit of these estimates. Mitchell *et al*. evaluated and calibrated STIM on both in-silico and controlled experimental data [13], showing that STIM can capture temporal trends of MOI by using surveillance data from Asembo, Kenya between 1996 to 2012.

The calibrations of STIM and StrainRecon revealed optimum hyperparameter values. The minimum viable resolution thresholds of STIM were strains above 5% proportion and *k* estimates of 5 or less; therefore, this study used these same thresholds, so strains below 5% proportion were discarded and MOI predictions considered only values in *k*∈[1,5]. STIM’s misfit threshold was set to 1.8e-7 for every sample, following earlier validation [13]; keeping the threshold parameter constant ensured that each sample was processed in a consistent manner, preserving trends across different samples and populations. The noise level parameter gamma of StrainRecon was set to 0.01.

### **Figure S1: Statistical analysis of temporal trends of MOI**

Statistical differences between multiple distributions were determined using the Kruskal-Wallis test for differentiation between three or more distributions, followed by Conover-Imam tests for differentiation between individual distributions [41][42]. These tests revealed significant differentiation between the MOI distributions of each year (Kruskal-Wallis *p*=9.27e-8) and significant differentiation between the distributions in any two years except for 2001-2012, 2001-2017, and 2012-2017(Figure S1).

**Figure S2: Statistical analysis to compare MOI between age groups by year.**

Conover-Iman test was used for comparing the MOIs between each age group, with Benjamini-Yekutieli FDR correction. Note that 1996 was excluded for analysis because all subjects in that year were in one group <=5 years old. The q-values indicate no significant differences in the MOIs between each age group in any other years.

### **Method S2: Relatedness Metrics**

#### Method S2-1: *F_ST_*

If $H_{t}$ is the gene diversity of the full population and $H_{s}$ is the within-population gene diversity, then *F_ST_* can be calculated using the following equation:

$$F_{ST}=\frac{H_{t} - H_{s}}{H_{t}}$$

Hierfstat was used for the calculations [52].

#### Method S2-2: IBD

The concept of IBD relatedness is based on the process by which strains cross over strands of DNA during reproduction. Each SNP site can be either IBD or not IBD. Whether or not two observed strains match at these SNP sites is a result of the genotyping error rate and whether those SNP sites are latently IBD. Whether or not a SNP site is latently IBD is, in turn, a result of whether strains match at that site and whether nearby SNP sites are IBD. Thus, the IBD for a pair of strains is modelled using a Hidden Markov Model. The hidden states of the model are whether or not each site is IBD, and the observed matches at each site inform these hidden states. A full IBD model for a population is then represented by three free parameters: the initial probabilities of being IBD and not-IBD, the transition probability of changing between IBD and not-IBD, and the emission probability of seeing identical or not-identical SNPs given the hidden state of being IBD or not-IBD. These parameters can be optimised using the maximum likelihood algorithm.

Calculations were performed using HmmIBD software [17][53]. HmmIBD uses the following parameters:

-the recombination rate (set to the default value for *Plasmodium falciparum*)

-the genotyping error rate (set to the default value of 0.1%)

-the allele frequencies for each value at each SNP (inferred from data)

-number of iterations (set to the default value of 5)

### **Method S3 and Figure S3: IBD Relatedness within and across years**

Figure S2-1 further confirms the low IBD-relatedness of the strain populations. The relatedness of the real populations was compared to that of a baseline of randomly generated strains. SNP allele values of strains in each year’s baseline were drawn from the distribution of SNP allele values of that year. Thus, the baseline eliminates the associations between SNP sites used by IBD while maintaining the underlying allele distributions. Comparing each pair of years to its baseline revealed no discernible difference, as confirmed using Kolmogorov-Smirnov (KS) non-parametric statistical tests for differentiation between distributions (KS-test, *p*>0.77 for each pair of years, shown in Figure S2-2). Thus, the strains in this study are no more related than those that would be obtained by random draws, suggesting that the studied malaria parasite populations are both highly diverse and well mixed.

### **Figure S4: Within-host IBD strain relatedness differentiation**

A Kruskal–Wallis one-way analysis of variance of the frequencies of within-host relatedness values for each year revealed significant differentiation between years (*p*=7.05e-6). Conover-Iman test *q*-values with two-stage non-negative false discovery rate (FDR) correction (Figure S3) indicate that 1996 and 2001 differed significantly from all other years, but there was no significant differentiation between 2007, 2012, or 2017.

### **Method S4 and Figure S5: Verification of relationship between within-host IBD and MOI**

Analyses conducted on two different models confirmed the significance of the inverse relationship between within-subject relatedness and MOI.

To determine the effect of the analysis itself, strains in the first baseline were shuffled between the subjects within each year. This preserved the strains and MOIs within each year but removed the relationship between strains within each subject. The results in Figure S5-1 show that the relationship between within-host relatedness and MOI in this baseline is weak and slightly positive, contrary to that of the original data. This difference suggests that the analysis had negligible effect on the discovered trends.

To determine the possible effect of StrainRecon’s limited accuracy in reconstructing low-proportion strains, the second baseline consisted only of the higher proportion strains of the original dataset. Specifically, the baseline consisted of two subsets: the strains over 10% proportion, and the strains over 20% proportion. This preserved the strains within the subjects but removed any low-proportion strains in the subjects, thus removing the effects of StrainRecon’s limited accuracy. The results in Figures S5-2 and S5-3 show that the strong inverse relationship observed between within-host relatedness and MOI was still present in each of these high-proportion datasets. This suggests that the limited accuracy of StrainRecon had negligible effects on the discovered trends.

### **Method S5: *H_e_* and within-population gene diversity *H_s_***

*H_e_* is equal to the average of the squares of the frequency of each allele at each SNP site, whereas *H_s_* is a modification of *H_e_* that corrects for systematic sampling biases recently discovered in *H_e_* calculations.

Define $p_{ij}$ as the probability of the locus *j* having haplotype *i* in a given population. Let *m* be the number of loci, so $1\leq j\leq m$.

Each locus can take values between 0 and *k*, representing possible allales. In the case of SNP barcodes, *k=1,* and the set of possible alleles is {0,1}. Note that $\sum_{i}^{k} p_{ij}=1$ for any *j*.

Define $\underline{{p_{i}}^{2}}=\frac{1}{m}\sum_{j=1}^{m} p_{ij}^{2}$ for any i.

The heterozygosity of a population over multiple loci is defined as:

$H_{e} = 1 - \frac{1}{m}\sum_{i}^{k} \sum_{j=1}^{m} p_{ij}^{2} = 1-\sum_{i}^{k} \underline{{p_{i}}^{2}}$

To calculate the within-population gene diversity *H_s_* for a population of haploids, let N be the total number of strains, n_i_ be the number of strains with haplotype i (note that $p_{i} =\frac{n_{i}}{N}$), and*ñ* be the harmonic mean of n_i_ over all i. Then the within-population gene diversity of a sample of haplotypes is

$$H_{s}=\frac{ñ}{(ñ-1)}\left[ 1 - \sum_{i}^{k} \underline{{p_{i}}^{2}} \right]$$

### **Method S6 and Table S1: Effective Population Size**

The effective population size is an estimator of the size of a population, providing a model from which trends in a population can be analysed. This study used NeEstimator v2.1 to calculate *N_e_* using two methods.

**Linkage Disequilibrium (LD)**: When the values of certain genes tend to occur together more than with others, it is called “linkage disequilibrium.” In a finite population with no selection bias, linkage disequilibrium occurs as a result of genetic drift. The intensity of the disequilibrium varies predictably with the size of the population; therefore, by measuring it in a sample population, one can estimate the size of the overall population [54].

**Temporal:** Imbalances in allele frequencies occur naturally over time as a result of genetic drift. The rate of these imbalances varies predictably with the size of the population, so given two samples of a population at different times, one can compare their allele frequencies and use that to estimate the size of the overall population. This study used Jorde and Ryman’s method using Plan II sampling for estimation [55]. Malaria generation timeframe was configured at 2 months [53][56].

**References**

1. Goudet JF. FSTAT (version 1.2): A computer program to calculate F-statistics. J Heredity. 1995;86:485-6.
2. Schaffner SF, Taylor AR, Wong W, Wirth DF, Neafsey DE. hmmIBD: software to infer pairwise identity by descent between haploid genotypes. Malar J. 2018;17:196.
3. Hill WG. Estimation of effective population size from data on linkage disequilibrium1. Genetics Research. 1981;38:209-16.
4. Jorde PE, Ryman N. Temporal allele frequency change and estimation of effective size in populations with overlapping generations. Genetics. 1995;139:1077-90.
5. Sisya TJ, Kamn’gona RM, Vareta JA, Fulakeza JM, Mukaka MF, Seydel KB, et al. Subtle changes in *Plasmodium falciparum* infection complexity following enhanced intervention in Malawi. Acta Trop. 2015;142:108-14.
