# Supplementary material for: Assessing the impact of temporal changes in transmission on Plasmodium falciparum strains in Asembo, western Kenya (1996–2017) using within-host metrics via 24-SNP barcodes
Source: Malar J. 2025 Dec 17;25:49. doi: 10.1186/s12936-025-05700-3 (PMC12829202; doi:10.1186/s12936-025-05700-3)
Supplement: Supplementary file 2 — Supplementary material 2. Table S1: Effective population sizes for all years. [file 12936_2025_5700_MOESM2_ESM.docx]

**Table S1: Effective population sizes for all years.**

| **Year** | **N_e_ (LD)** | **Year 2** | ***N_e_ (Temporal)** |
| --- | --- | --- | --- |
| 1996 | 240.7 | 2001 | 2573.3 |
| 2001 | 237.1 | 2007 | 2645.3 |
| 2007 | 136.5 | 2012 | 2472.9 |
| 2012 | 210.6 | 2017 | 2805.4 |
| 2017 | 292.1 |  |  |

Calculations are performed using the Linkage Disequilibrium (N_e_ (LD)) method, and the Temporal method (*N_e_ (Temporal)).
