# Supplementary figures and images for: Assessing the impact of temporal changes in transmission on Plasmodium falciparum strains in Asembo, western Kenya (1996–2017) using within-host metrics via 24-SNP barcodes
Source: Malar J. 2025 Dec 17;25:49. doi: 10.1186/s12936-025-05700-3 (PMC12829202; doi:10.1186/s12936-025-05700-3)

**Figure S1**


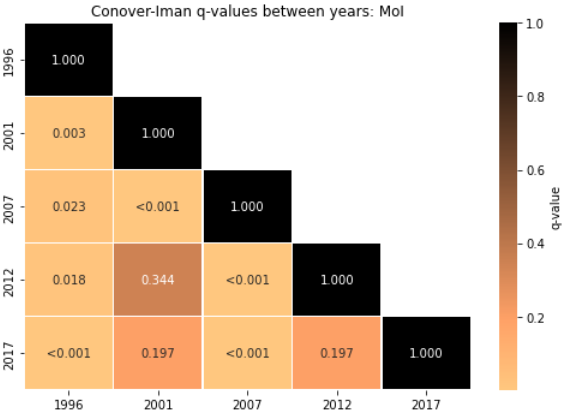

Supplement: Supplementary file 3 — Supplementary material 3. Fig. S1 Comparison of MOI between years. Conover-Imam q-values are given of pairwise comparisons across years. [file 12936_2025_5700_MOESM3_ESM.docx]

**Figure S2**


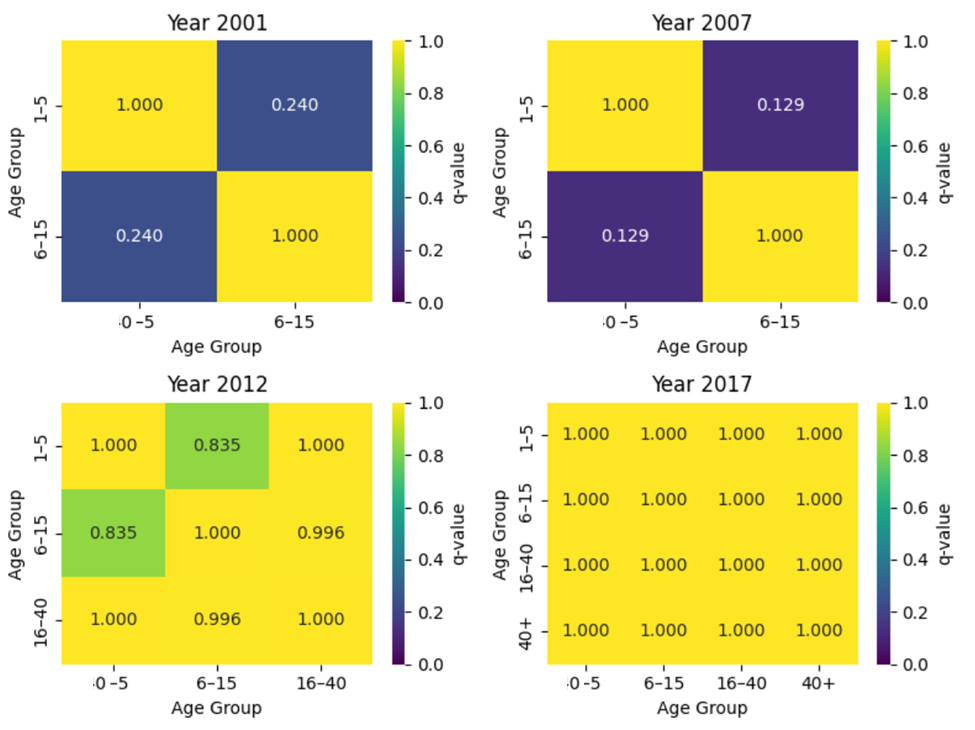

Supplement: Supplementary file 4 — Supplementary material 4. Fig. S2 Comparison of MOI between age groups by year. Conover-Iman test was used for comparing the MOIs between each age group, with Benjamini-Yekutieli FDR correction. Note that 1996 was excluded for analysis because all subjects in that year were in one group <=5 years old. The q-values indicate no significant differences in the MOIs between each age group in each year. [file 12936_2025_5700_MOESM4_ESM.docx]

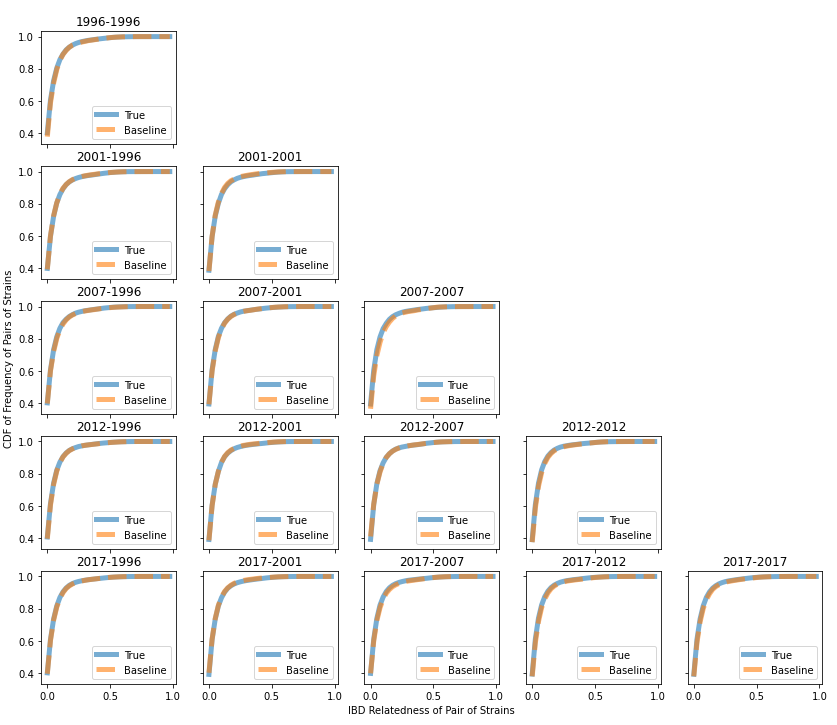


**Figure S3-1**


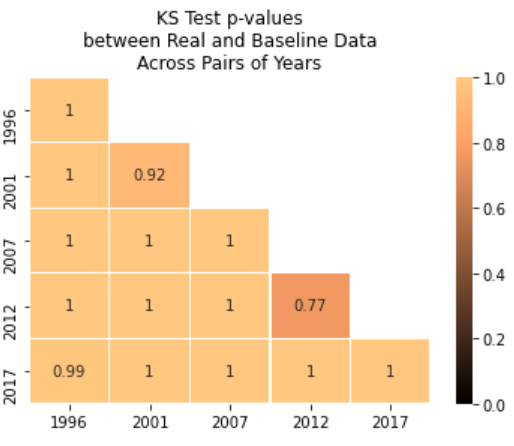

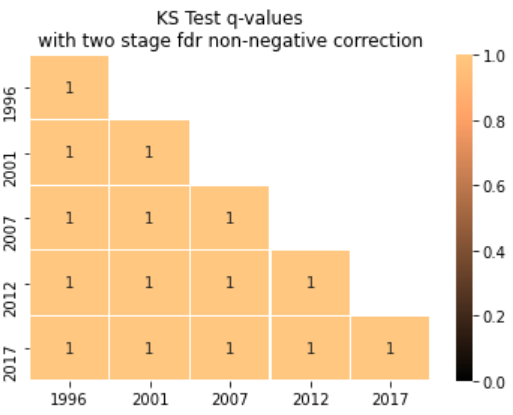


**Figure S3-2**

Supplement: Supplementary file 5 — Supplementary material 5. Fig. S3 1 IBD strain-relatedness compared to baseline. CDF of frequencies of IBD strain relatedness are shown within years on the diagonal and across years off the diagonal. Each pair was compared against its baseline pair, where each year’s baseline was made from strains randomly drawn from the distribution of SNPs. 2 KS tests comparing the relatedness of strains within and across years to their baselines. The p-values of these comparisons (left), as well as the q-values obtained by performing two-stage non-negative false discovery rate corrections (right), do not exhibit statistically significant differences between the real distributions and their baselines. [file 12936_2025_5700_MOESM5_ESM.docx]

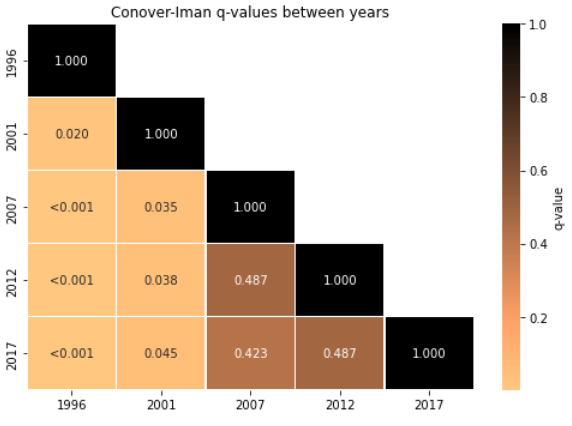


**Figure S4**

Supplement: Supplementary file 6 — Supplementary material 6. Fig. S4 Conover-Iman statistical q-values with FDR for differentiation of within-host relatedness between years. Lower values indicate more significant differentiation between years, and higher values indicate less significant differentiation. 1996 and 2001 show greater differentiation from other years than 2007, 2012, and 2017. [file 12936_2025_5700_MOESM6_ESM.docx]

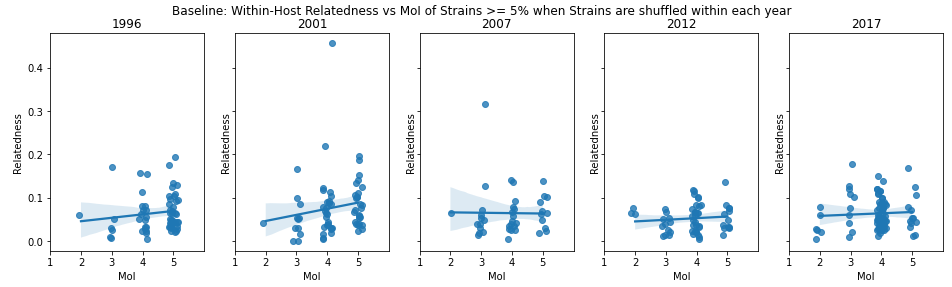


**Figure S5-1**

**
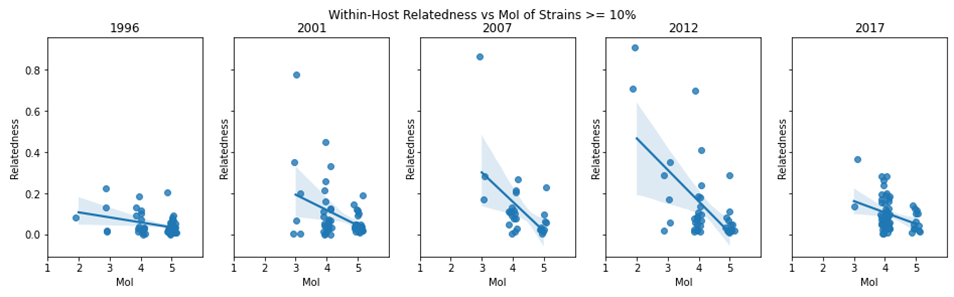
**

**Figure S5-2**


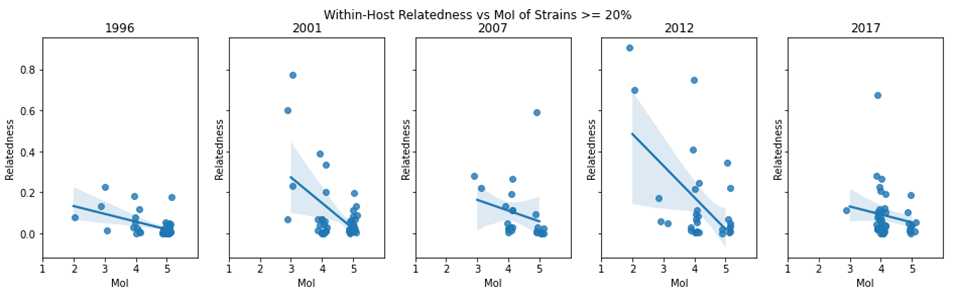


**Figure S5-3**

Supplement: Supplementary file 7 — Supplementary material 7. Fig. S5 1 Analysis of the relationship between baseline within-host relatedness and MOI with strains shuffled across subjects within each year. Slopes of MOI vs within-host relatedness show none of the trends seen in empirical data, suggesting that the trends found are a result of true patterns in the samples analysed and are not an artefact of the analysis itself. 2 Analysis of the relationship between within-host relatedness and MOI in high-proportion (>=10%) strains. The result of analysing only strains exceeding 10% proportions is shown. Results reflect the trends seen in empirical data, suggesting that the trends found are a result of true patterns in the samples analysed and are not an artefact of the analysis itself. 3 Analyses of the relationship between within-host relatedness and MOI in high-proportion (>=20%) strains. The results of analysing only strains exceeding 20% proportions are shown. Results reflect the trends seen in empirical data, suggesting that the trends found are a result of true patterns in the samples analysed and are not an artefact of the analysis itself. [file 12936_2025_5700_MOESM7_ESM.docx]
